# Distinct patterns of genetic overlap among multimorbidities revealed with trivariate MiXeR

**DOI:** 10.1186/s13073-025-01528-3

**Published:** 2025-09-29

**Authors:** Alexey A. Shadrin, Guy Hindley, Espen Hagen, Nadine Parker, Markos Tesfaye, Piotr Jaholkowski, Zillur Rahman, Gleda Kutrolli, Vera Fominykh, Srdjan Djurovic, Olav B. Smeland, Kevin S. O’Connell, Dennis van der Meer, Oleksandr Frei, Ole A. Andreassen, Anders M. Dale

**Affiliations:** 1 Centre for Precision Psychiatry, Division of Mental Health and Addiction, Oslo University Hospital, and Institute of Clinical Medicine, University of Oslo, Oslo, Norway; 2 KG Jebsen Centre for Neurodevelopmental Disorders, University of Oslo and Oslo University Hospital, Oslo, Norway; 3 Institute of Psychiatry, Psychology and Neuroscience, King’s College London, 16 De Crespigny Park, London, SE5 8AB UK; 4 Department of Clinical Science, University of Bergen, Bergen, Norway; 5 Department of Medical Genetics, Oslo University Hospital, Oslo, Norway; 6 School of Mental Health and Neuroscience, Faculty of Health, Medicine and Life Sciences, Maastricht University, Maastricht, the Netherlands; 7 Center for Bioinformatics, Department of Informatics, University of Oslo, 0316 Oslo, Norway; 8 Department of Radiology, University of California, San Diego, La Jolla, CA 92093 USA; 9 Multimodal Imaging Laboratory, University of California San Diego, La Jolla, CA 92093 USA; 10 Department of Psychiatry, University of California, San Diego, La Jolla, CA USA; 11 Department of Neurosciences, University of California San Diego, La Jolla, CA 92093 USA; 12 Visiting Address: Section for Precision Psychiatry, Building 48, Oslo University Hospital, Ullevål, Kirkeveien 166, PO Box 4956, 0424 Nydalen, Oslo Norway; 13 Visiting and Postal Address: J. Craig Venter Institute, 4120 Capricorn Lane, La Jolla, CA 92037 USA; 14 Visiting Address: Section for Precision Psychiatry, Building 49, Oslo University Hospital, Ullevål, Kirkeveien 166, PO Box 4956, 0424 Nydalen, Oslo Norway

**Keywords:** Multimorbidity, Complex phenotypes, The pattern of genetic overlap, GWAS, Trivariate MiXeR

## Abstract

**Background:**

Multimorbidities are a global health challenge. Accumulating evidence indicates that overlapping genetic architectures underlie comorbid complex human traits and disorders. This can be quantified for a pair of phenotypes using various techniques. Still, the pattern of genetic overlap between three distinct complex phenotypes, which is important for understanding multimorbidities, has not been possible to quantify.

**Methods:**

Here, we present and validate the novel trivariate MiXeR tool, which disentangles the pattern of genetic overlap between three complex phenotypes using summary statistics from genome-wide association studies. Our simulations show that trivariate MiXeR can reliably reconstruct different patterns of genetic overlap and estimate the proportions of genetic overlap between three phenotypes.

**Results:**

We found substantial genetic overlap between gastro-intestinal and brain diseases supporting a genetic basis of the gut-brain axis—the pattern consistent with pairwise analysis. However, the pattern of genetic overlap between three diverse cardiometabolic and renal health indicators and three immune-linked disorders revealed a much larger genomic component shared between all phenotypes than expected from separate pairwise analyses. This suggests the existence of core pathways underlying distinct but related chronic conditions.

**Conclusions:**

Overall, trivariate MiXeR offers a novel and efficient tool for investigating patterns of genetic overlap among three complex phenotypes. This contributes to a better understanding of genetic relationships between complex traits and disorders, potentially providing new insights into the mechanisms underlying common multimorbidities. Trivariate MiXeR is freely available at https://github.com/precimed/mix3r.

**Supplementary Information:**

The online version contains supplementary material available at 10.1186/s13073-025-01528-3.

## Background

Multimorbidity, or suffering from two or more chronic illnesses [1], is a global health challenge [2]. There is limited knowledge about the underlying mechanisms beyond the individual diseases. Many chronic illnesses are complex human traits and are highly polygenic, with thousands of associated loci discovered to date [3]. Most functional genetic loci affect multiple phenotypes, resulting in intricate patterns of genetic overlap [4]. Characterizing patterns of overlapping genetic architecture between pairs of phenotypes has generated key discoveries in human genetics and revealed the shared genetic underpinnings for a wide range of common human diseases [5]. However, there are no methods to specifically characterize genetic overlap beyond two complex traits or diseases. A more complete understanding of the complex genetic relationships between multiple traits and disorders can help to elucidate shared genetics and highlight suggestive mechanisms of multimorbidities leading to improvements in disease classification, diagnostics, and patient-centered treatment and care [6].

The causal mixture model (MiXeR) set of tools can model genetic architecture and characterize genetic overlap between complex traits and diseases. Univariate MiXeR [3] quantifies characteristics of the genetic architecture for complex phenotypes, including polygenicity, which reflects the number of genetic variants influencing a phenotype. Bivariate MiXeR [7], in turn, quantifies the overlapping polygenic component between two phenotypes regardless of the effect directions of the shared variants. This represented an important advance in attempts to decode the shared genetic architecture between pairs of traits since the previous standard measure, genetic correlation, can only detect genetic overlap in pairs of phenotypes where the bulk of variants have either concordant or discordant directions of effects [8]. A balanced mixture of concordant and discordant effects will provide a genetic correlation close to zero, similar to a pair of genetically disjoint phenotypes, making these two scenarios indistinguishable [9]. Bivariate MiXeR can characterize the relationship between various pairs of phenotypes beyond genetic correlation, effectively capturing shared genetics with mixed directions of effects [5, 10–12].

However, no existing methods can estimate genetic overlap across three phenotypes. Apart from trivial cases of non-overlapping or completely overlapping phenotypes, trivariate overlap cannot accurately be reconstructed by analyzing each pair of phenotypes within a triad (i.e., three bivariate analyses). For a given combination of three bivariate overlaps, the maximum entropy distribution of the trivariate overlap can be conceptualized as the center of all possible trivariate overlap distributions. However, indirect reconstruction under this naïve assumption of overlapping parts may lead to erroneous estimates. Thus, estimating genetic overlap among triads of phenotypes can help reveal important aspects of genetic overlap associated with different sets of phenotypes, tissue types, or biological mechanisms. This is important for the identification of shared and unique genetic architecture underlying specific multimorbidities.

Several methods exist that allow for the combination of GWAS summary statistics for more than two phenotypes, exploiting the overlapping nature of their genetic architectures. These pleiotropy-motivated methods cover various actively developing domains of statistical genetics analysis, including MTAG [13] and sfFDR [14] for improved discovery of genetic loci, mvSuSiE [15] and CAFEH [16] for fine-mapping, mr.mash-rss [17] and MT-Bayes [18] for polygenic prediction, and GenomicSEM [19] for modeling joint covariance structure. While some of these methods implicitly model genetic overlap across multiple phenotypes (for example, the multivariate Bayesian regression method designed for improved prediction introduced by Cheng et al. [18]), they are intended for different purposes and have not been presented or tested in the context of dissecting the pattern of genetic overlap independent of genetic correlation. Therefore, we did not perform a formal comparison between trivariate MiXeR and these methods.

Here, we present trivariate MiXeR, which disentangles the pattern of polygenic overlap among three complex phenotypes using summary statistics from genome-wide association studies (GWASs). We first conduct a series of simulations covering diverse scenarios of genetic overlap among three phenotypes and demonstrate that the tool can reliably reconstruct different patterns of trivariate genetic overlap. Next, we apply trivariate MiXeR to determine the genetic architecture of triads of complex phenotypes involved in multimorbidities for which epidemiological studies suggest shared causal pathways. This revealed that the pattern of genetic overlap between genetic generalized epilepsy (GGE), irritable bowel syndrome (IBS), and attention deficit hyperactivity disorder (ADHD) was as expected from bivariate MiXeR. For type 2 diabetes (T2D), estimated glomerular filtration rate (eGFR) and high-density lipoprotein (HDL), the estimated overlap among all three phenotypes was twice as large as the naïve expectation. A larger shared genetic component between all three phenotypes was also found for ulcerative colitis (UC), multiple sclerosis (MS) and psoriasis (PS). Our analyses demonstrate non-trivial patterns of trivariate genetic overlap that are substantially different from naïvely expected patterns derived from three bivariate analyses. This suggests the presence of core shared molecular mechanisms underlying distinct but related phenotypes.

## Methods

### Trivariate MiXeR model

The method extends the bivariate MiXeR model [7] for the case of three phenotypes, leaving the basic assumptions of the model unchanged. Briefly, an additive model of genetic effects is considered. In the univariate analysis, the direct (not induced by linkage disequilibrium) effect ${\beta }_{j}$ of the *j*^th^ variant on a phenotype is modeled as a mixture of null and phenotype-influencing (non-null) components characterized by two parameters: the proportion of variants influencing the phenotype (polygenicity, $\pi \in [\text{0,1}]$) and the variance of their effect sizes (discoverability, ${\sigma }^{2}$):

$${\beta }_{j}=\left\{\begin{array}{c}0, 1-\pi \\ N\left(0,{\sigma }^{2}\right), \pi \end{array}\right.$$

where $N\left(0,{\sigma }^{2}\right)$ is a normal distribution with zero mean and ${\sigma }^{2}$ variance.

In a joint analysis of three phenotypes ($i=1, 2, 3$), a fraction of variants may affect all three phenotypes (${\pi }_{123}$), other variants may affect a pair of phenotypes but not the third phenotype (${\pi }_{12}$, ${\pi }_{13}$, ${\pi }_{23}$), some variants might be phenotype-specific (${\pi }_{1},{\pi }_{2},{\pi }_{3}$) while most variants are expected to have no effect on any phenotype (${\pi }_{0}=1-{\pi }_{1}-{\pi }_{2}-{\pi }_{3}-{\pi }_{12}-{\pi }_{13}-{\pi }_{23}-{\pi }_{123}$). We assume all variants for a given phenotype follow the same distribution of effect sizes (with corresponding discoverabilities ${\sigma }_{1}^{2}$, ${\sigma }_{2}^{2}$, ${\sigma }_{3}^{2}$), regardless of their effects on the other two phenotypes. Genetic correlations are modeled by introducing correlations of effect sizes within each of three pairwise overlaps (${\rho }_{12},{\rho }_{13},{\rho }_{23}$). With these assumptions, the trivariate distribution of direct effects of the *j*^th^ variant is modeled as a mixture of eight components:

$$\left(\begin{array}{c}{\beta }_{1j}\\ {\beta }_{2j}\\ {\beta }_{3j}\end{array}\right)=\left\{\begin{array}{c}\begin{array}{c}\overline{0 }, {\pi }_{0}\\ N\left(\overline{0 },{{\varvec{\Sigma}}}_{1}\right), {\pi }_{1}\end{array}\\ \begin{array}{c}\begin{array}{c}N\left(\overline{0 },{{\varvec{\Sigma}}}_{2}\right), {\pi }_{2}\\ N\left(\overline{0 },{{\varvec{\Sigma}}}_{3}\right), {\pi }_{3}\end{array}\\ \begin{array}{c}N\left(\overline{0 },{{\varvec{\Sigma}}}_{12}\right), {\pi }_{12}\\ N\left(\overline{0 },{{\varvec{\Sigma}}}_{13}\right), {\pi }_{13}\end{array}\\ \begin{array}{c}N\left(\overline{0 },{{\varvec{\Sigma}}}_{23}\right), {\pi }_{23}\\ N\left(\overline{0 },{{\varvec{\Sigma}}}_{123}\right), {\pi }_{123}\end{array}\end{array}\end{array}\right.$$

where $$\overline{0 }=\left(\begin{array}{c}0\\ 0\\ 0\end{array}\right)$$ and $${{\varvec{\Sigma}}}_{1}=\left(\begin{array}{ccc}{\sigma }_{1}^{2}& 0& 0\\ 0& 0& 0\\ 0& 0& 0\end{array}\right)$$, $${{\varvec{\Sigma}}}_{2}=\left(\begin{array}{ccc}0& 0& 0\\ 0& {\sigma }_{2}^{2}& 0\\ 0& 0& 0\end{array}\right)$$, $${{\varvec{\Sigma}}}_{3}=\left(\begin{array}{ccc}0& 0& 0\\ 0& 0& 0\\ 0& 0& {\sigma }_{3}^{2}\end{array}\right)$$, $${{\varvec{\Sigma}}}_{12}=\left(\begin{array}{ccc}{\sigma }_{1}^{2}& {\rho }_{12}{\sigma }_{1}{\sigma }_{2}& 0\\ {\rho }_{12}{\sigma }_{1}{\sigma }_{2}& {\sigma }_{2}^{2}& 0\\ 0& 0& 0\end{array}\right)$$, $${{\varvec{\Sigma}}}_{13}=\left(\begin{array}{ccc}{\sigma }_{1}^{2}& 0& {\rho }_{13}{\sigma }_{1}{\sigma }_{3}\\ 0& 0& 0\\ {\rho }_{13}{\sigma }_{1}{\sigma }_{3}& 0& {\sigma }_{3}^{2}\end{array}\right)$$, $${{\varvec{\Sigma}}}_{23}=\left(\begin{array}{ccc}0& 0& 0\\ 0& {\sigma }_{2}^{2}& {\rho }_{23}{\sigma }_{2}{\sigma }_{3}\\ 0& {\rho }_{23}{\sigma }_{2}{\sigma }_{3}& {\sigma }_{3}^{2}\end{array}\right)$$,

$${{\varvec{\Sigma}}}_{123}=\left(\begin{array}{ccc}{\sigma }_{1}^{2}& {\rho }_{12}{\sigma }_{1}{\sigma }_{2}& {\rho }_{13}{\sigma }_{1}{\sigma }_{3}\\ {\rho }_{12}{\sigma }_{1}{\sigma }_{2}& {\sigma }_{2}^{2}& {\rho }_{23}{\sigma }_{2}{\sigma }_{3}\\ {\rho }_{13}{\sigma }_{1}{\sigma }_{3}& {\rho }_{23}{\sigma }_{2}{\sigma }_{3}& {\sigma }_{3}^{2}\end{array}\right)$$ are covariance matrices of multivariate normal distributions corresponding to the different phenotype-influencing components**.**

The joint signed association test statistics (z-score) of the *j*^th^ variant is then given by:

$$\left(\begin{array}{c}{z}_{1j}\\ {z}_{2j}\\ {z}_{3j}\end{array}\right)=\sum_{k=1}^{M}\sqrt{{h}_{k}}{r}_{jk}\left(\begin{array}{c}\sqrt{{N}_{1j}}{\beta }_{1k}\\ {\sqrt{{N}_{2j}}\beta }_{2k}\\ {\sqrt{{N}_{3j}}\beta }_{3k}\end{array}\right)+\epsilon$$

where ${N}_{ij}$ ($i=1, 2, 3$) is the sample size of the GWAS for the *i*^th^ phenotype and *j*^th^ variant, ${h}_{k}$ is the heterozygosity of variant *k*, $M$ is the number of variants in linkage disequilibrium (LD) with the variant *k*, ${r}_{jk}$ is the Pearson’s correlation coefficient between the genotypes of the *j*^th^ and *k*^th^ variants (quantifying LD), and $\epsilon \sim N\left(\overline{0 },{{\varvec{\Sigma}}}_{0}\right)$ is a normally distributed vector of residuals with covariance matrix

$${{\varvec{\Sigma}}}_{0}=\left(\begin{array}{ccc}{\sigma }_{01}^{2}& {\rho }_{012}{\sigma }_{01}{\sigma }_{02}& {\rho }_{013}{\sigma }_{01}{\sigma }_{03}\\ {\rho }_{012}{\sigma }_{01}{\sigma }_{02}& {\sigma }_{02}^{2}& {\rho }_{023}{\sigma }_{02}{\sigma }_{03}\\ {\rho }_{013}{\sigma }_{01}{\sigma }_{03}& {\rho }_{023}{\sigma }_{02}{\sigma }_{03}& {\sigma }_{03}^{2}\end{array}\right),$$

where ${\sigma }_{0i}^{2}$ ($i=1, 2, 3$) is a residual variance of the *i*^th^ phenotype and ${\rho }_{0ij}$ ($i,j=1, 2, 3$) is a correlation between residuals of the *i*^th^ and *j*^th^ phenotypes. Nineteen parameters of the model $\left({\pi }_{1},{\pi }_{2},{\pi }_{3},{\pi }_{12},{\pi }_{13},{\pi }_{23},{\pi }_{123},{\sigma }_{1},{\sigma }_{2},{\sigma }_{3},{\sigma }_{01},{\sigma }_{02},{\sigma }_{03},{\rho }_{12},{\rho }_{13},{\rho }_{23},{\rho }_{012},{\rho }_{013},{\rho }_{023}\right)$ are estimated by maximizing the likelihood of the z-scores observed in the GWAS summary statistics using a step-wise procedure. First, three univariate analyses are performed to estimate univariate polygenicities (${\pi }_{1}^{u},{\pi }_{2}^{u},{\pi }_{3}^{u}$), discoverabilities (${\sigma }_{1},{\sigma }_{2},{\sigma }_{3}$) and residual variances (${\sigma }_{01},{\sigma }_{02},{\sigma }_{03}$) for each of the three phenotypes. Then the bivariate analyses are performed to estimate pairwise genetic overlaps (${\pi }_{12}^{b},{\pi }_{13}^{b},{\pi }_{23}^{b}$), correlations of effect sizes within each of the three pairwise overlaps (${\rho }_{12},{\rho }_{13},{\rho }_{23}$) and correlations between residuals (${\rho }_{012},{\rho }_{013},{\rho }_{023}$) for each of the three pairs of phenotypes with univariate parameters fixed to the values obtained at the univariate step. Finally, the genetic overlap between all three phenotypes (${\pi }_{123}$) is estimated with both univariate and bivariate parameters fixed to the values obtained in the univariate and bivariate steps. Phenotype pair-specific polygenicities can then be calculated as:

$${\pi }_{12}= {\pi }_{12}^{b}-{\pi }_{123},$$

$${\pi }_{13}= {\pi }_{13}^{b}-{\pi }_{123},$$

$${\pi }_{23}= {\pi }_{23}^{b}-{\pi }_{123},$$

and phenotype-specific polygenicities can be calculated as:

$${\pi }_{1}={\pi }_{1}^{u}-{\pi }_{12}-{\pi }_{13}-{\pi }_{123},$$

$${\pi }_{2}={\pi }_{2}^{u}-{\pi }_{12}-{\pi }_{23}-{\pi }_{123},$$

$${\pi }_{3}={\pi }_{3}^{u}-{\pi }_{13}-{\pi }_{23}-{\pi }_{123}.$$

Nomenclature of pattern proportion parameters () is illustrated in (row 3, column B).

The model is not restricted to locus lead variants or variants with a significance level above a certain cutoff; it models the full spectrum of variants and accounts for LD between them.

Univariate, bivariate, and trivariate log-likelihood functions are implemented using numerical integration of the characteristic function applying a trapezoidal rule with fixed step size as described previously [20].

### Simulation setup

**Fig. 1:**
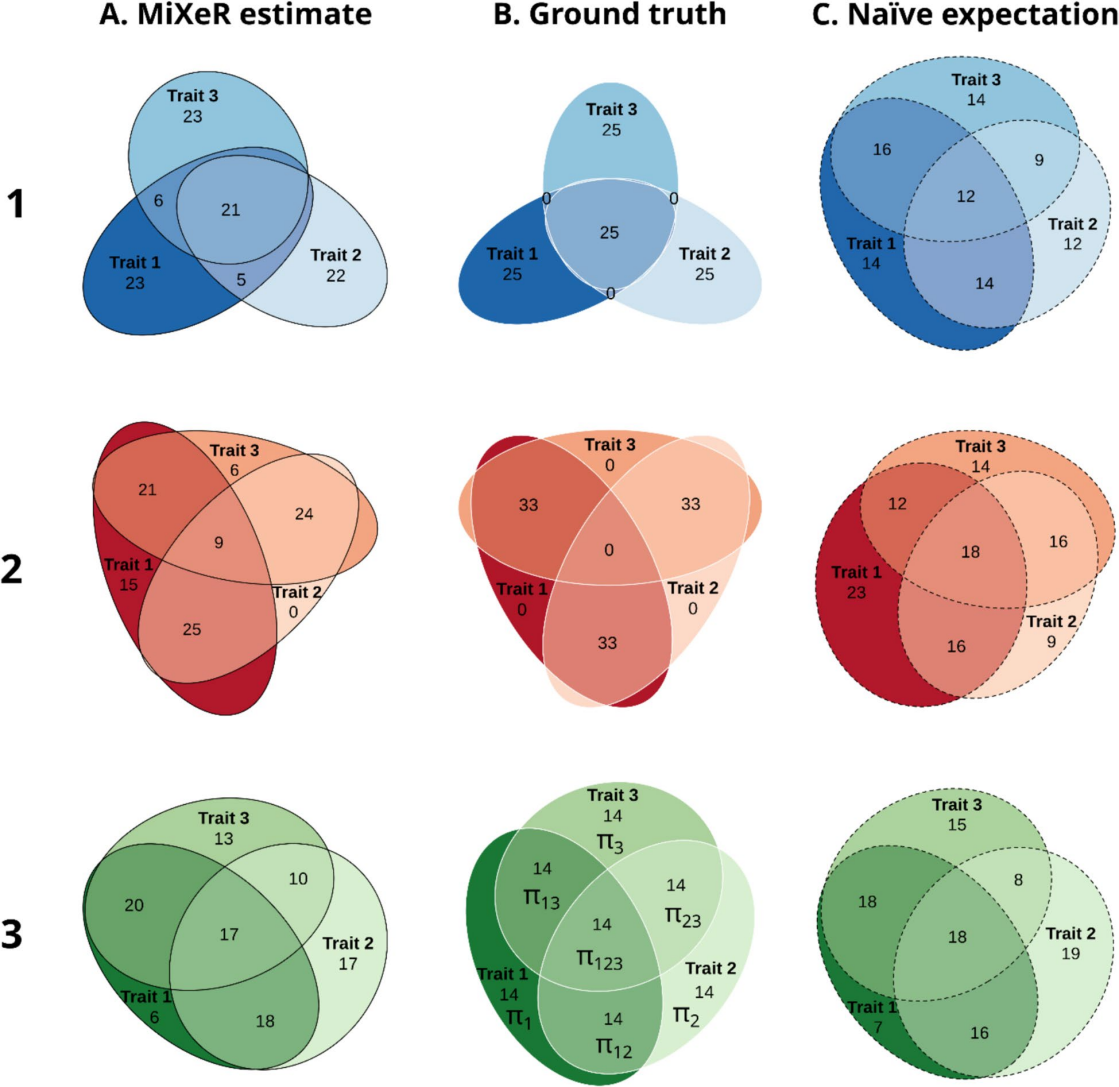
Simulated data. Three different scenarios of genetic overlap in simulated data (rows) estimated by trivariate MiXeR (column A, solid black outline), compared to the theoretical true pattern of the simulated overlap (column B, solid white outline) and the overlap pattern expected for the estimated bivariate overlaps under naïve assumption of maximum entropy (column C, dashed black outline). Row 1 (blue colors): “Core” scenario; Row 2 (red colors): “Ring” scenario; Row 3 (green colors): “Equilibrium” scenario (see `Methods` for further details). For each simulation scenario (within each row), for every area of each diagram, its percentage with respect to the combined total area of all three phenotypes in the estimated diagram (column A) is shown (rounded to the closest integer), i.e., percentages within each diagram in the column A add up to 100 and percentages within each row are directly comparable with those shown in column A. Since percentages in column C are also given with respect to the combined total area of the corresponding diagram in column A, the sum of percentages in column C is not necessarily equal to 100. For each scenario, phenotypes were simulated independently; therefore, “Trait 1,” “Trait 2,” and “Trait 3” in row 1 are not the same as “Trait 1,” “Trait 2,” and “Trait 3” in row 2 or in row 3 of the figure, respectively. The middle diagram in the bottom row (row 3, column B) shows the nomenclature for proportions of areas within the pattern (${\pi }_{1},{\pi }_{2},{\pi }_{3},{\pi }_{12},{\pi }_{13},{\pi }_{23},{\pi }_{123}$) used throughout the text

To validate the method and to test its ability to discriminate different scenarios of genetic overlap, we performed a series of analyses with simulated data. GWAS summary statistics for simulations were generated based on participants randomly selected from the UK Biobank using 100,000 unrelated (defined by 22,020 data-field) white British (defined by 22,006 data-field) individuals and version 3 of the imputed genetic data. UK Biobank data was obtained under accession number 27412. Autosomal variants with minor allele frequency above 0.1%, genotype missingness below 10%, imputation info score above 0.8 and passing Hardy–Weinberg equilibrium test at *p* = 10^−10^, totaling 12,926,691 variants were included in the analysis. A set of quantitative phenotypes with equal polygenicity ($\pi =0.002$), equal SNP-heritability ($h2=0.4$) and different patterns of genetic overlap were generated using the SIMU tool [21]. For each phenotype, a given number of phenotype-influencing variants ($n=\text{25,853}\approx \text{12,926,691}*0.002$) were selected at random. The effect sizes for the selected variants were sampled from a standard normal distribution and scaled to obtain the predefined SNP-heritability. For each individual analyzed, a quantitative synthetic phenotype was then generated as the sum of allelic effects over all phenotype-influencing variants complemented by a certain proportion of random Gaussian noise (representing environmental effects) required to keep the predefined level of heritability. Association analysis was performed using PLINK2 [22, 23] with sex, age and the first 10 genetic principal components included as covariates. Three simulation scenarios were considered:

1. “Core”: only triple overlap, i.e., all overlapping variants are shared between all three phenotypes, for each phenotype half of the phenotype-influencing variants also influence both of the other phenotypes. ${\pi }_{1}={\pi }_{2}={\pi }_{3}={\pi }_{123}, {\pi }_{12}={\pi }_{13}={\pi }_{23}=0$. Presented in Fig. 1, row 1.
2. “Ring”: no triple overlap, for each phenotype half of the phenotype-influencing variants also influence one of the remaining two phenotypes and the second half influences another phenotype, ${\pi }_{12}={\pi }_{13}={\pi }_{23}, {\pi }_{1}={\pi }_{2}={\pi }_{3}={\pi }_{123}=0$. Presented in Fig. 1, row 2.
3. “Equilibrium”: balanced mixture of all three phenotypes, ${\pi }_{1}={\pi }_{2}={\pi }_{3}={\pi }_{12}={\pi }_{13}={\pi }_{23}={\pi }_{123}$. Presented in Fig. 1, row 3.

For each triad of phenotypes, sixteen independent optimization runs were performed to maximize the likelihood of the GWAS z-scores observed in different subsets of 500,000 randomly selected variants. We then calculated the median across these sixteen runs for each polygenicity parameter ($\pi_{S}^{\textrm{median}} = \underset{\textrm{run median}}(\pi_{S}), S\in \left\{1, 2, 3, 12, 13, 23, 123\right\}, \textrm{run}=1,\ldots ,16$) and find the run with the smallest deviation from the median overlap pattern. A Euler diagram for this run is then presented both for the simulated data and for the real data analysis. Of note, the pattern constituted from the median polygenicities (${\pi }_{S}^{\text{median}}, S\in \left\{1, 2, 3, 12, 13, 23, 123\right\}$) is not guaranteed to be feasible itself, since proportions in the overlap of three phenotypes are constrained, as described in the “Naïve expectation” section below, and these constraints are not necessarily fulfilled for the median proportions across multiple patterns which are themselves feasible.

### Genome-wide association studies (GWAS) data

For the analysis of the real data, we used publicly available GWAS summary statistics on nine phenotypes: GGE (6,952 cases, 42,436 controls) [24], IBS (24,735 cases, 77,149 controls) [25], ADHD (38,691 cases, 186,843 controls) [12], T2D (80,154 cases, 853,816 controls) [26], eGFR (567,460 individuals) [27], HDL (1,244,580 individuals) [28], UC (12,366 cases, 33,609 controls) [29], MS (14,802 cases, 26,703 controls) [30] and PS (965 cases, 437,420 controls) [31] (FinnGen, release 11, phenotypic code L12_PSORIASIS). All summary statistics were based on individuals of European ancestry. We analyzed patterns of genetic overlap for three triads: (1) GGE, IBS, and ADHD, (2) T2D, eGFR, and HDL, and (3) UC, MS, and PS. The selection of these triads intends to illustrate that the method may provide insights into a wide spectrum of traits and disorders affecting different organs and tissues.

### Naïve expectation

Given univariate estimates of polygenicity for three phenotypes (${\pi }_{1}^{u},{\pi }_{2}^{u},{\pi }_{3}^{u}$), three bivariate estimates of genetic overlap between these phenotypes (${\pi }_{12}^{b},{\pi }_{13}^{b},{\pi }_{23}^{b}$) obtained in univariate and bivariate analyses for the triad of phenotypes the genetic overlap between these three phenotypes is constrained by bounds${\pi }_{123}\in \left[{\pi }_{123}^{\text{min} },{\pi }_{123}^{\text{max}}\right]$, where ${\pi }_{123}^{\text{min} }=\text{max}\left(0,{\pi }_{12}^{b}+{\pi }_{13}^{b}-{\pi }_{1}^{u},{\pi }_{12}^{b}+{\pi }_{23}^{b}-{\pi }_{2}^{u},{\pi }_{13}^{b}+{\pi }_{23}^{b}-{\pi }_{3}^{u}\right)$ and ${\pi }_{123}^{\text{max }}=\text{min}\left({\pi }_{12}^{b},{\pi }_{13}^{b},{\pi }_{23}^{b}\right)$. Without any prior knowledge about genetic relationships between analyzed phenotypes, a naïve expectation about the value of ${\pi }_{123}$ follows the principle of maximum entropy:

$${\pi }_{123}^{\text{na} \ddot{i} \text{ve} }=\text{argmax} H\left({\pi }_{123}\right)=\underset{{\pi }_{123}\in \left[{\pi }_{123}^{\text{min} },{\pi }_{123}^{\text{max} }\right]}{\text{argmax}}\sum_{p=({\pi }_{1},{\pi }_{2},{\pi }_{3},{\pi }_{12},{\pi }_{13},{\pi }_{23},{\pi }_{123})}-p*\text{log}(p)$$

In other words for the given univariate (${\pi }_{1}^{u},{\pi }_{2}^{u},{\pi }_{3}^{u}$) and bivariate (${\pi }_{12}^{b},{\pi }_{13}^{b},{\pi }_{23}^{b}$) polygenicities, the naïve trivariate polygenicity (${\pi }_{123}^{\text{na} \ddot{i} \text{ve}}$) is selected so that among all the possible distributions (${\pi }_{1},{\pi }_{2},{\pi }_{3},{\pi }_{12},{\pi }_{13},{\pi }_{23},{\pi }_{123}$) with the constraint’s${\pi }_{123}\in \left[{\pi }_{123}^{\text{min} },{\pi }_{123}^{\text{max}}\right]$, the probability distribution (${\pi }_{1}^{\text{na} \ddot{i} \text{ve} },{\pi }_{2}^{\text{na} \ddot{i} \text{ve} },{\pi }_{3}^{\text{na} \ddot{i} \text{ve} },{\pi }_{12}^{\text{na} \ddot{i} \text{ve} },{\pi }_{13}^{\text{na} \ddot{i} \text{ve} },{\pi }_{23}^{\text{na} \ddot{i} \text{ve} },{\pi }_{123}^{\text{na} \ddot{i} \text{ve}}$) has maximum entropy, where

$${\pi }_{12}^{\text{na} \ddot{i} \text{ve} }= {\pi }_{12}^{b}-{\pi }_{123}^{\text{na} \ddot{i} \text{ve} },$$

$${\pi }_{13}^{\text{na} \ddot{i} \text{ve} }= {\pi }_{13}^{b}-{\pi }_{123}^{\text{na} \ddot{i} \text{ve} },$$

$${\pi }_{23}^{\text{na} \ddot{i} \text{ve} }= {\pi }_{23}^{b}-{\pi }_{123}^{\text{na} \ddot{i} \text{ve} },$$

$${\pi }_{1}^{\text{na} \ddot{i} \text{ve} }={\pi }_{1}^{u}-{\pi }_{12}^{\text{na} \ddot{i} \text{ve} }-{\pi }_{13}^{\text{na} \ddot{i} \text{ve} }-{\pi }_{123}^{\text{na} \ddot{i} \text{ve} },$$

$${\pi }_{2}^{\text{na} \ddot{i} \text{ve} }={\pi }_{2}^{u}-{\pi }_{12}^{\text{na} \ddot{i} \text{ve} }-{\pi }_{23}^{\text{na} \ddot{i} \text{ve} }-{\pi }_{123}^{\text{na} \ddot{i} \text{ve} },$$

$${\pi }_{3}^{\text{na} \ddot{i} \text{ve} }={\pi }_{3}^{u}-{\pi }_{13}^{\text{na} \ddot{i} \text{ve} }-{\pi }_{23}^{\text{na}\ddot{i} ve }-{\pi }_{123}^{\text{na} \ddot{i} \text{ve} }.$$

are the naïvely expected phenotype pair-specific and phenotype-specific polygenicities. Of note, polygenicities for the naïve expectation (${\pi }_{1}^{\text{na} \ddot{i} \text{ve} },{\pi }_{2}^{\text{na} \ddot{i} \text{ve} },{\pi }_{3}^{\text{na} \ddot{i} \text{ve} },{\pi }_{12}^{\text{na} \ddot{i} \text{ve} },{\pi }_{13}^{\text{na} \ddot{i} \text{ve} },{\pi }_{23}^{\text{na}\ddot{i} \text{ve} },{\pi }_{123}^{\text{na} \ddot{i} \text{ve}}$) are calculated based on univariate (${\pi }_{1}^{u},{\pi }_{2}^{u},{\pi }_{3}^{u}$) and bivariate (${\pi }_{12}^{b},{\pi }_{13}^{b},{\pi }_{23}^{b}$) polygenicities estimated by the trivariate MiXeR model; therefore, the univariate polygenicity of each phenotype as well as the shared polygenicity of each phenotypic pair are the same for the naïvely expected pattern and the pattern estimated by the trivariate MiXeR, i.e.,${\pi }_{12}^{\text{na} \ddot{i} \text{ve} }+ {\pi }_{123}^{\text{na} \ddot{i} \text{ve} }={\pi }_{12}^{\text{model}}+ {\pi }_{123}^{\text{model} }= {\pi }_{12}^{b}$, and similar for other bivariate and univariate polygenicities.

### Linkage disequilibrium reference panel

Simulations were performed using an LD reference panel constructed based on 10,000 randomly selected unrelated white British individuals from the UK Biobank. Quality control procedures for variants were identical to those described in the “Simulations” section leaving 12,926,691 variants in the LD reference panel. PLINK 1.9 [22, 32] was applied to estimate r2 coefficients within each autosome using –ld-window 1,000,000 –ld-window-kb 20,000 –ld-window-r2 0.01 parameters. The resulting text files were then processed to produce input files in the format required by MiXeR, using the scripts provided in the code repository [33].

Real data analyses were performed using an LD reference panel constructed based on 489 European individuals from 1000 Genomes Phase 3 data provided by [34]. Variants with minor allele frequency below 3% were removed, and remaining 6,739,595 variants were used for calculation of r2 coefficients within each autosome using PLINK 1.9 with –ld-window 1,000,000 –ld-window-kb 30,000 –ld-window-r2 0.05 parameters.

### Trivariate MiXeR model implementation

Building on the same assumptions as the bivariate MiXeR model, we have extended the framework to include three phenotypes modeling the trivariate distribution of genetic effects as a mixture of eight components. Compared to the bivariate MiXeR tool, the code for log-likelihood estimation was re-implemented using the numerical integration of the characteristic function. This facilitated more stable convergence of the optimization algorithm and reduced fluctuations in the estimates caused by the random sampling approach applied in the bivariate MiXeR v1.3 implementation [35], at the cost of substantially increased computational burden. To cope with the increased computational demand, performance-critical parts have been accelerated using graphics processing units (GPUs). Trivariate MiXeR code can only be deployed on machines with GPUs supporting NVIDIA CUDA, which are now commonly available on high-performance computing (HPC) facilities or cloud computing facilities.

The trivariate MiXeR tool is implemented in Python mainly using numpy [36], scipy [37] and pandas [38] packages, while the “numba” just-in-time (JIT) compiler [39] is used to translate the Python and numpy-based routines into machine code. Performance-critical steps rely on the availability of a GPU, NVIDIA CUDA. Overlapping patterns are visualized using the “eulerr” R package [40]. The execution environment with all dependencies can be created using Conda [41] mamba, or micromamba [42].

Input parameters for the analysis can be tuned and provided in the configuration file in JSON format. An example configuration file showing parameters is available in the code repository (https://github.com/precimed/mix3r/blob/main/config_t2d_hdl_egfr_oct30_1.paper.json). Important parameters used in all presented analyses are as follows: maf_thresh = 0.05—z-scores of the variants with minor allele frequency (MAF) below 5% were not used for optimization (MAF is estimated from the same genotypes which are used to construct the LD reference panel); info_thresh = 0.8—z-scores of the variants with imputation INFO score below 0.8 are not used for optimization, if input sumstats do not contain INFO column the filter is ignored; z_thresh = 32—z-scores with absolute value larger than 32 were not used for optimization; exclude_regions = 6:25,000,000–34000000—variants from the major histocompatibility complex (chr6:25,000,000–34000000, hg19 genomic guild) were not used for optimization; do_pruning = true, r2_prune_thresh = 0.8—prior to optimization, variants were randomly pruned with allelic correlation threshold r2 < 0.8; n_random = 300,000—a subset of 300 K variants was randomly selected for optimization from all variants surviving random pruning; rand_prune_seed = 1—a seed for the generator of the pseudo-random numbers (controls both random pruning and random sub-setting of variants), changing this parameter while keeping all other parameters unchanged allows the user to repeat the analysis with a different subset of variants. In this study rand_prune_seed = 1, …, 16 were used to produce 16 independent runs for each triad of phenotypes.

### Runtime and memory usage

Most computations in trivariate MiXeR are performed on a GPU, which keeps central processing unit (CPU) requirements moderate. Optimization of random-access memory (RAM) usage is achieved through efficient data flow operations. For example, a complete LD panel is never fully loaded into RAM; instead, it is processed as a memory-mapped file on disk.

Analyses presented in the manuscript were conducted in a computational environment using 4 CPU cores, 32 GB of RAM, and a single NVIDIA A100-PCIe card with 40 GB of memory. In our additional tests, a minimal configuration with 2 CPU cores and 16 GB of RAM was sufficient to analyze three summary statistics, each with approximately 10 million variants, while further increasing the number of CPUs or available memory did not affect the runtime. The choice of GPU, however, had an important effect, summarized in Table 1.

**Table 1:** Runtime assessments for different GPU models

GPU model	Runtime mean (Std), hours
A100-PCIe (40 GB RAM)	2.575 (0.075)
V100-PCIe (32 GB RAM)	3.425 (0.090)
P100-PCIe (16 GB RAM)	6.215 (0.545)
RTX 3090-PCIe (24 GB RAM)	42.217 (3.016)

We measured runtimes on four different GPU accelerators: the NVIDIA A100-PCIe (40 GB memory), V100-PCIe (32 GB memory), P100-PCIe (16 GB memory), and RTX 3090-PCIe (24 GB memory). For these runs, we used three synthetic summary statistics generated for the “ Equilibrium” simulation scenario described in the “Simulation setup” section of the manuscript.

For each GPU model, we performed 16 independent runs using 300,000 randomly selected subsets of variants for optimization. The average runtime and corresponding standard deviation over these 16 runs for each GPU model are presented in Table 1.

## Results

### Simulations and validation

Our simulations demonstrated that MiXeR was able to capture the true patterns of genetic overlap for all three simulated scenarios (Fig. 1). For “core” (Fig. 1, row 1) and “ring” (Fig. 1, row 2) scenarios, the trivariate MiXeR model accurately captured the disproportional pattern of overlap in the simulated datasets (column A), while the result expected for the estimated bivariate overlaps under the naïve assumption of the maximum entropy probability distribution (column C) revealed substantial deviation from the true simulated pattern (column B). For the balanced “equilibrium” scenario (Fig. 1, row 3) the true pattern (column B) followed the maximum entropy principle; thus, the naïve expectation (column C) should be no worse than the pattern reconstructed by trivariate MiXeR (column A). As can be seen in this case, the naïve pattern and the pattern reconstructed by the trivariate MiXeR model were very similar, illustrating adequate model fit. Estimates of all model parameters for 16 independent MiXeR runs for “core,” “ring,” and “equilibrium” scenarios are shown in Additional file 1:Table S1, Table S2, and Table S3, respectively.

### Analysis of sensitivity to misspecification of linkage disequilibrium

We assessed the sensitivity of the model to the misspecification of the LD structure. For this purpose, we used simulated UK Biobank-based GWAS summary statistics generated for the “Equilibrium” scenario described in the “Simulation setup” subsection of the “Methods” section. The analysis was performed using two different LD panels: the UK Biobank and the 1000 Genomes-based panel described in the “Linkage disequilibrium reference panel” subsection of the “Methods.”

For each LD reference panel, 64 independent runs were performed using 300,000 randomly selected subsets of variants for optimization. A vector of polygenicity parameters (${\pi }_{1}^{u},{\pi }_{2}^{u},{\pi }_{3}^{u}$, ${\pi }_{12}^{b},{\pi }_{13}^{b},{\pi }_{23}^{b},{\pi }_{123}$) from each run based on one LD reference panel was then correlated with the vector of polygenicity parameters from each run based on another LD reference panel. In total, 64 × 64 = 4096 correlation values were computed, resulting in a mean correlation of *r* = 0.969 (SD = 0.021). Additionally, we estimated correlations between all runs produced with the same LD reference panel, yielding 32 × 63 = 2016 correlation values for each LD reference panel. This resulted in a mean *r* = 0.977 (SD = 0.015) for the UK Biobank-based panel and a mean *r* = 0.972 (SD = 0.020) for the 1000 Genomes-based panel.

These results show that while there is a slightly higher discrepancy across runs between different LD reference panels than within each panel, the results between different panels are highly consistent. This suggests that the unspecific European LD reference panel based on populations available in the 1000 Genomes Project might provide an adequate characterization of the genome-wide pattern of genetic overlap when applied to GWAS summary statistics from a specific European population.

### Real-world multimorbidity data

**Fig. 2:**
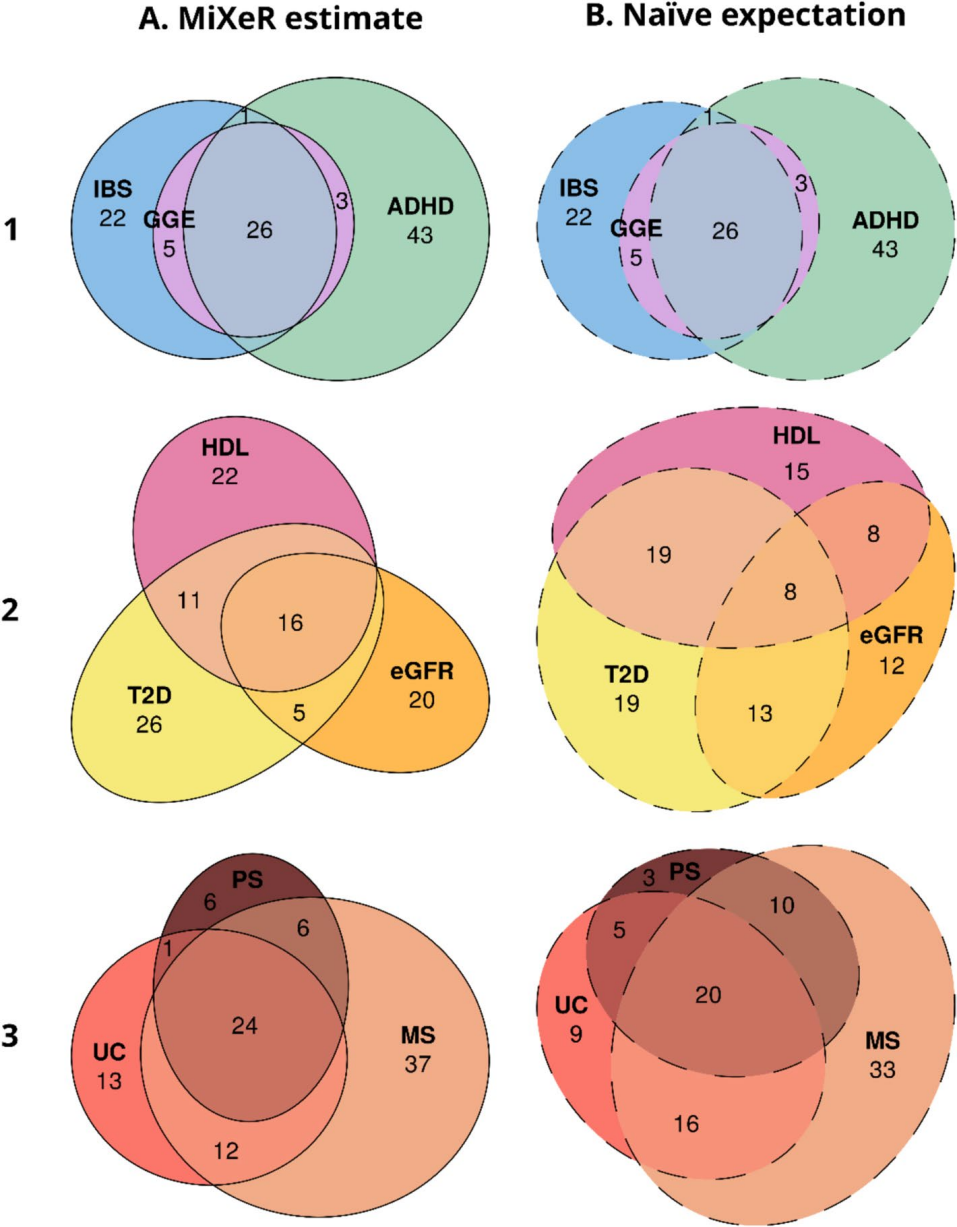
Real data. Genetic overlap between complex human traits and disorders estimated by trivariate MiXeR (column A) compared to naïve expectation following the principle of maximum entropy (column B). The pattern of genetic overlap between genetic generalized epilepsy (GGE), irritable bowel syndrome (IBS) and attention deficit hyperactivity disorder (ADHD) (row 1), type 2 diabetes (T2D), estimated glomerular filtration rate (eGFR) and high-density lipoprotein (HDL) (row 2), ulcerative colitis (UC), multiple sclerosis (MS) and psoriasis (PS) (row 3). For each triad of phenotypes (within each row), for every area of each diagram, its percentage with respect to the combined total area of all three phenotypes in the estimated diagram (column A) is shown (rounded to the closest integer), i.e., percentages within each diagram in column A add up to 100 and percentages within each row are directly comparable. Since percentages in column B are also given with respect to the combined total area of the corresponding diagram in column A, the sum of percentages in column B is not necessarily equal to 100. Areas of ellipses are proportional to the polygenicities of respective phenotypes. The size of each Euler diagram is scaled separately and therefore areas in different diagrams are not comparable

We applied trivariate MiXeR to GWAS summary statistics on three triads of phenotypes with different levels of polygenicity. We then compared the pattern of genetic overlap estimated by trivariate MiXeR (Fig. 2, column A) with the pattern expected for the given bivariate overlaps under a naïve expectation of the maximum entropy probability distribution (Fig. 2, column B).

### Psychiatric, neurological, and gastrointestinal diseases

The first analysis (Fig. 2, row 1), included three highly polygenic phenotypes, with ADHD being the most polygenic (univariate polygenicity, ${\pi }^{u}$ = 3.0E-3, constituting 73% of the combined total area) followed by IBS (${\pi }^{u}$ = 2.2E-3, 54%) and GGE (${\pi }^{u}$ = 1.4E-3, 34%). This analysis suggests that variants shared between all three phenotypes (26% of the combined total area) closely match the variants influencing GGE with only small fraction of the latter being outside the overlap (3% and 5% shared with only ADHD and IBS, respectively). For this triad, the naïve expectation from three separate bivariate analyses provides the same pattern of the overlap as the trivariate analysis (Fig. 2, row 1, column B). All model parameters are presented in Additional file 1: Table S4.

### Cardiometabolic and renal health indicators

Compared to the first analysis, the second analysis (Fig. 2, row 2) included less polygenic phenotypes: T2D (${\pi }^{u}$ = 8.5E-4), eGFR (${\pi }^{u}$ = 5.8E-4), and HDL (${\pi }^{u}$ = 7.2E-04). The modeled overlap pattern for this triad of phenotypes resembled the “core” simulation scenario (Fig. 1, row 1). The estimated overlap among all three phenotypes (16% of the combined total area) was twice as large as the naïve expectation (8%). Similarly, estimated phenotype-specific areas were larger than expected, constituting 26% (the naïve expectation 19%), 20% (12%), and 22% (15%) of the combined total area for T2D, eGFR, and HDL, respectively. The increase in size of the triple overlap and phenotype-specific areas in MiXeR-derived patterns is compensated by the reduction of the phenotype pair-specific areas comprising 5% (13%) for T2D and eGFR, 0% (8%) for eGFR and HDL, and 11% (19%) for T2D and HDL pairs respectively. All model parameters are presented in Additional file 1: Table S5.

### Immune-linked diseases

The third triad included phenotypes with the lowest polygenicities among the presented analyses (Fig. 2, row 3): ulcerative colitis (${\pi }^{u}$ = 1.7E-4), multiple sclerosis (${\pi }^{u}$ = 2.7E-4), and psoriasis (${\pi }^{u}$ = 1.3E-4). Similar to the second analysis, this analysis revealed a “core”-like pattern of overlap, with the component shared between all three phenotypes and the phenotype-specific components larger than their naïve expectation counterparts, while phenotype pair-specific fractions were smaller. However, in this analysis, deviation from the naïve expectation was less pronounced than in the analysis of type 2 diabetes, estimated glomerular filtration rate, and high-density lipoprotein. All model parameters are presented in Additional file 1: Table S6.

The three examples illustrate the range of observed discrepancies between naïve expectations based on bivariate MiXeR and patterns estimated by trivariate MiXeR, which demonstrate the importance of applying trivariate analysis when studying the genetic architecture of multimorbidities.

## Discussion

We have developed and validated the trivariate MiXeR tool to disentangle the pattern of genetic overlap among three complex phenotypes using genome-wide summary statistics. We show that the genetic overlap underlying trios of brain (neurology, psychiatry) and gastrointestinal diseases is as expected from bivariate analysis, while the genetic overlap across cardiometabolic and renal health indicators and across immune-related diseases is larger than expected from respective bivariate analyses. These results demonstrate that there are novel patterns of trivariate genetic architecture among complex diseases and traits that cannot be reliably deduced from pairwise overlap estimates. This may contribute to a better understanding of the genetic etiology of multimorbidities and their biological underpinnings, which can inform the development of better preventive and treatment options.

Pathophysiological mechanisms behind GGE, ADHD, and IBS are largely unknown, with some indications of genetic overlap [43, 44]. Our analysis suggests that the genetic underpinnings of GGE are comprised within the shared genetic architecture of IBS and ADHD, while both ADHD and IBS also reveal substantial disorder-specific genetic components. While perhaps unexpected, this finding could be explained by the fact that core neurotransmitter systems maintaining excitatory/inhibitory balance play a critical role in GGE and constitute a substantial part of its heritable component [24, 45]. Dysregulated excitatory/inhibitory balance is also a prominent feature of ADHD, altering brain function during neurodevelopment [46], and IBS, in which the balance of excitatory/inhibitory neurotransmission affects the functional components of the gut-brain axis [47]. Nevertheless, ADHD and IBS have important differences both aetiologically and clinically, not least that ADHD predominantly affects higher brain functions while IBS affects the gastrointestinal system, helping to explain their notable unique genetic components.

Our trivariate MiXeR analysis of T2D, high-density lipoprotein, and a measure of renal function (eGFR) demonstrates substantial polygenic overlap between these three phenotypes, a pattern of overlap which differed from that expected from bivariate MiXeR results. Such extensive overlap indicates the existence of core pathways involved in these related but distinct indicators of chronic ill health. In clinical and epidemiological studies, T2D has been associated with structural changes and abnormal function of high-density lipoprotein [48], which may impact renal function and increase the risk of kidney disease [49, 50]. In particular, the “core” pattern of genetic overlap between these phenotypes may suggest that there are subgroups of individuals which are at risk for this cluster of multimorbidity, while other individuals may be more likely to develop specific conditions [51].

Our analysis of genetic overlap between UC, PS, and MS revealed a large number of disease-specific variants while the shared component was predominantly within the triple overlap. These findings are consistent with the hypothesis that there is a common genetic basis for immune-linked diseases [52] with a core combination of genetic mutations driving them [53]. Disturbance of these hypothetical core immune processes might activate the breakdown in immune tolerance [52] necessary to trigger any of these diseases, which may also underlie the well-known phenomenon of multimorbidity in immune-linked diseases [54]. The subsequent developmental trajectory of a given autoimmune disease may then be driven, in part, by disease-specific genetic factors, supported by the phenotype-specific regions on the trivariate Euler diagram. Determining and disentangling core and specific genetic factors for immune diseases may provide valuable insights into key immune pathways and cell types involved in disease mechanisms, with potential for drug target development.

We have demonstrated how trivariate MiXeR can estimate the proportions of polygenic overlap among diverse human traits and diseases, highlighting patterns of genetic overlap that could not be deduced from bivariate MiXeR. The simulations showed that the tool can reliably reconstruct different patterns of genetic overlap. Pairwise genetic overlaps between multiple phenotypes have been extensively studied and have provided valuable insights into the shared and phenotype-specific genetic architectures of different traits and disorders [5, 55, 56]. However, estimating pairwise genetic overlaps among three phenotypes does not provide a complete picture of the genetic overlap among those three phenotypes. We show that the trivariate MiXeR model can dissect the pattern of genetic overlap among three complex phenotypes, using GWAS summary statistics, and reveal patterns of overlap that are distinct from naïve expectations based on bivariate MiXeR. Our clinical examples demonstrate that trivariate MiXeR can elucidate situations where a triad of phenotypes can overlap disproportionately, providing novel insights into the variability in overlapping genetic underpinnings among those phenotypes.

In clinical practice, non-random aggregation of diseases emerges as multimorbidity, representing a steadily growing challenge to the entire medical system [57]. However, current efforts to understand the underlying causes and biological disease mechanisms have been limited by a single disease approach. Here we show that trivariate MiXeR can capture the unique genetic landscape of co-occurring chronic diseases. This will help us to be more systematic in our approach to multimorbidity, and ultimately develop more patient-centered treatment and care [6].

In certain situations, it may be tempting and informative to study the genetic overlap between more than three phenotypes. While extending the mathematical model underlying MiXeR to accommodate more than three phenotypes is conceptually feasible, practical implementation using the current framework presents challenges due to prohibitive computational costs, primarily related to the complexity of numerical integration involved in evaluating the cost function. Furthermore, the simultaneous analysis of many phenotypes may face significant interpretability challenges, as the number of potential overlap combinations increases exponentially with the number of phenotypes examined. Even if the technical challenges outlined above are resolved, interpreting the intricate pattern that arises from the interactions of multiple phenotypes may still be difficult due to insufficient resolution of the estimates.

Trivariate MiXeR has the same limitations as the univariate and bivariate MiXeR models [3, 7]. The underlying model is sensitive to LD structure estimation, and the reliability of parameter estimates depends on the statistical power of the input GWAS summary statistics. MiXeR can have difficulty distinguishing pleiotropy from confounding by strong LD. In simulations for the bivariate model, we found that this could lead to a subtle overestimation of shared polygenicity in some scenarios, although the bias did not exceed 10% in well-powered analyses [7]. Our sensitivity analysis revealed a high consistency between the results produced for well-powered UK Biobank-based GWAS summary statistics using either UK Biobank or 1000 Genomes-based LD reference panels. However, these findings may not be generalized to more genetically distant populations. The model makes a few simplifying assumptions, including the uniform distribution of phenotype-influencing variants across the genome and the effect size’s independence from allele frequency, LD, and location in the genome. These assumptions may be violated to different degrees for different phenotypes, making the model less suitable for some phenotypes than for others. Analysis of phenotypes combining a handful of strong genetic effects with a weak polygenic background (for example Alzheimer’s disease [3]) may be sensitive to the selection of variants used for parameter fitting. To assess the stability of optimization convergence and the robustness of obtained results, trivariate MiXeR performs multiple independent runs using different subsets of variants and carefully assesses the variation of parameter estimates across runs to evaluate the model’s suitability for each set of phenotypes.

## Conclusions

We have developed and validated the trivariate MiXeR model and demonstrated its utility in disentangling the pattern of genetic overlap between three complex diseases and traits. Our results show that there are novel patterns of trivariate genetic architecture of complex diseases and traits that are substantially different from pairwise overlap-based expectations. We provide the tool implementing the model along with convenient ways of visualizing the results. The code is complemented with documentation and examples of how to use it (https://github.com/precimed/mix3r). The trivariate MiXeR tool can help to provide insight into the genetic relationship across complex human phenotypes to better understand the underlying mechanisms of multimorbidities.

## Supplementary Information

Additional file 1. File includes six tables with parameter estimates for 16 independent trivariate MiXeR runs: one table for each of the three simulation scenarios and one table for each of the three triads of real-world phenotypes.

## Data Availability

The individual-level genotypes used for simulations in this study are available from UK Biobank. The data used in this study were obtained under accession number 27412. Researchers who want to access this resource must register with UK Biobank by completing the registration form in the Access Management System at https://www.ukbiobank.ac.uk. Individual-level genotypes of 489 European individuals from 1000 Genomes Phase 3 are available at https://zenodo.org/records/7768714 (DOI: https://doi.org/10.5281/zenodo.7768714) [34]. All GWAS summary statistics used in the study are publicly available: GGE (https://ftp.ebi.ac.uk/pub/databases/gwas/summary_statistics/GCST90271001-GCST90272000/GCST90271612/GCST90271612.tsv.gz) [24], IBS (https://ftp.ebi.ac.uk/pub/databases/gwas/summary_statistics/GCST90243001-GCST90244000/GCST90243958/GCST90243958_buildGRCh37.tsv) [25], ADHD (https://figshare.com/ndownloader/files/40036684) [12], T2D (“DIAMANTE-EUR.sumstat.txt.gz” file from https://personal.broadinstitute.org/ryank/DIAMANTE.sumstats.zip) [26], eGFR (https://ckdgen.imbi.uni-freiburg.de/files/Wuttke2019/20171017_MW_eGFR_overall_EA_nstud42.dbgap.txt.gz) [27], HDL (https://csg.sph.umich.edu/willer/public/glgc-lipids2021/results//ancestry_specific/HDL_INV_EUR_HRC_1KGP3_others_ALL.meta.singlevar.results.gz) [28], UC (https://ftp.sanger.ac.uk/pub/project/humgen/summary_statistics/human/2016–11-07/uc_build37_45975_20161107.txt.gz) [29], MS summary statistics data can be obtained after request to IMSGC data access committee at https://imsgc.net [30], PS summary statistics data can be downloaded after completing the registration form by following the instructions outlined at https://finngen.gitbook.io/documentation/r11/data-download [31]. The trivariate MiXeR tool and auxiliary scripts, including source code, documentation and examples of use are available at https://github.com/precimed/mix3r [33].

## Abbreviations

GWAS: Genome-wide association study
GGE: Genetic generalized epilepsy
IBS: Irritable bowel syndrome
ADHD: Attention deficit hyperactivity disorder
T2D: Type 2 diabetes
eGFR: Estimated glomerular filtration rate
HDL: High-density lipoprotein
UC: Ulcerative colitis
MS: Multiple sclerosis
PS: Psoriasis
LD: Linkage disequilibrium
GPU: Graphics processing unit
