# Supplementary material for: Distinct patterns of genetic overlap among multimorbidities revealed with trivariate MiXeR
Source: Genome Med. 2025 Sep 29;17:106. doi: 10.1186/s13073-025-01528-3 (PMC12482798; doi:10.1186/s13073-025-01528-3)
Supplement: Supplementary file 1 — Additional file 1. File includes six tables with parameter estimates for 16 independent trivariate MiXeR runs: one table for each of the three simulation scenarios and one table for each of the three triads of real-world phenotypes. [file 13073_2025_1528_MOESM1_ESM.docx]

| **Table S1.** Parameter estimates for 16 independent MiXeR runs for the "core" simulated scenario.  The first column show the index of the optimization run (1-16), with the run having the smallest deviation from the median overlap pattern (as described in the methods section) marked with asterisk. Other columns show univariate polygenicities ($\pi_{1}^{u},\pi_{2}^{u},\pi_{3}^{u}$), discoverabilities ($\sigma_{1},\sigma_{2},\sigma_{3}$) and residual variances ($\sigma_{01},\sigma_{02},\sigma_{03}$) for Trait 1, Trait 2 and Trait 3 respectively; pairwise (bivariate) genetic overlaps ($\pi_{12}^{b},\pi_{13}^{b},\pi_{23}^{b}$), correlations of effect sizes within each of the three pairwise overlaps ($\rho_{12},\rho_{13},\rho_{23}$) and correlations between residuals ($\rho_{012},\rho_{013},\rho_{023}$) for Trait 1 and Trait 2, Trait 1 and Trait 3 and Trait 2 and Trait 3 pairs respectively; the genetic overlap between all three phenotypes ($\pi_{123}$). | | | | | | | | | | | | | | | | | | | |
| --- | --- | --- | --- | --- | --- | --- | --- | --- | --- | --- | --- | --- | --- | --- | --- | --- | --- | --- | --- |
| Index | $\pi_{1}^{u}$ | $\sigma_{2}$ | $\sigma_{01}$ | $\pi_{2}^{u}$ | $\sigma_{2}$ | $\sigma_{02}$ | $\pi_{3}^{u}$ | $\sigma_{3}$ | $\sigma_{03}$ | $\pi_{12}^{b}$ | $\rho_{12}$ | $\rho_{012}$ | $\pi_{13}^{b}$ | $\rho_{13}$ | $\rho_{013}$ | $\pi_{23}^{b}$ | $\rho_{23}$ | $\rho_{023}$ | $\pi_{123}$ |
| 1 | 2.23E-03 | 8.40E-05 | 1.039 | 1.81E-03 | 1.04E-04 | 1.029 | 2.07E-03 | 9.01E-05 | 1.026 | 1.03E-03 | 0.000 | 0.000 | 1.08E-03 | 0.009 | -0.012 | 8.86E-04 | 0.053 | 0.003 | 8.86E-04 |
| 2 | 2.20E-03 | 8.63E-05 | 1.034 | 2.01E-03 | 1.00E-04 | 1.017 | 1.91E-03 | 9.81E-05 | 1.031 | 1.07E-03 | 0.000 | 0.000 | 1.03E-03 | 0.003 | -0.008 | 8.28E-04 | 0.068 | 0.002 | 8.28E-04 |
| 3 | 2.30E-03 | 8.56E-05 | 1.025 | 1.85E-03 | 1.03E-04 | 1.028 | 1.92E-03 | 9.96E-05 | 1.028 | 1.14E-03 | 0.000 | 0.001 | 1.02E-03 | 0.005 | -0.013 | 7.18E-04 | 0.157 | -0.004 | 7.18E-04 |
| 4 | 2.13E-03 | 9.07E-05 | 1.031 | 1.98E-03 | 9.90E-05 | 1.022 | 2.09E-03 | 9.18E-05 | 1.024 | 9.64E-04 | 0.000 | 0.000 | 1.16E-03 | 0.003 | -0.009 | 8.67E-04 | 0.055 | 0.002 | 8.67E-04 |
| 5 | 2.27E-03 | 8.50E-05 | 1.032 | 1.94E-03 | 1.02E-04 | 1.023 | 1.93E-03 | 9.63E-05 | 1.034 | 1.14E-03 | -0.004 | 0.005 | 1.11E-03 | 0.003 | -0.008 | 8.29E-04 | 0.106 | 0.000 | 8.29E-04 |
| 6 | 2.13E-03 | 8.84E-05 | 1.037 | 1.87E-03 | 1.05E-04 | 1.021 | 1.91E-03 | 9.72E-05 | 1.031 | 1.09E-03 | 0.000 | 0.000 | 1.00E-03 | 0.002 | -0.005 | 8.04E-04 | 0.096 | 0.001 | 6.45E-04 |
| 7 | 2.10E-03 | 9.00E-05 | 1.035 | 1.86E-03 | 1.04E-04 | 1.024 | 1.99E-03 | 9.25E-05 | 1.030 | 1.01E-03 | 0.000 | 0.000 | 1.05E-03 | 0.002 | -0.007 | 7.72E-04 | 0.049 | 0.004 | 7.72E-04 |
| 8 | 2.21E-03 | 8.80E-05 | 1.028 | 1.88E-03 | 1.02E-04 | 1.029 | 1.95E-03 | 9.53E-05 | 1.031 | 1.10E-03 | 0.001 | 0.004 | 1.01E-03 | 0.001 | -0.001 | 5.85E-04 | 0.208 | 0.000 | 5.85E-04 |
| 9 | 2.19E-03 | 8.81E-05 | 1.028 | 1.85E-03 | 1.00E-04 | 1.032 | 1.98E-03 | 9.27E-05 | 1.033 | 1.22E-03 | 0.000 | 0.000 | 9.99E-04 | 0.002 | -0.006 | 8.49E-04 | 0.059 | 0.002 | 8.49E-04 |
| 10 | 1.96E-03 | 1.00E-04 | 1.030 | 1.85E-03 | 1.05E-04 | 1.025 | 1.90E-03 | 9.79E-05 | 1.029 | 9.67E-04 | 0.000 | 0.000 | 9.25E-04 | 0.003 | -0.008 | 7.45E-04 | 0.097 | 0.000 | 7.45E-04 |
| 11 | 2.21E-03 | 8.58E-05 | 1.034 | 1.88E-03 | 1.02E-04 | 1.028 | 2.00E-03 | 9.49E-05 | 1.025 | 1.18E-03 | -0.004 | 0.005 | 1.11E-03 | 0.003 | -0.010 | 8.22E-04 | 0.091 | 0.001 | 8.22E-04 |
| 12 | 2.02E-03 | 9.83E-05 | 1.029 | 1.90E-03 | 1.04E-04 | 1.019 | 2.07E-03 | 9.30E-05 | 1.024 | 1.04E-03 | 0.000 | 0.000 | 1.11E-03 | 0.004 | -0.013 | 8.08E-04 | 0.089 | 0.001 | 8.08E-04 |
| 13 | 2.11E-03 | 9.28E-05 | 1.030 | 1.88E-03 | 1.02E-04 | 1.025 | 2.05E-03 | 9.57E-05 | 1.023 | 9.90E-04 | 0.000 | 0.000 | 1.06E-03 | 0.002 | -0.008 | 8.82E-04 | 0.047 | 0.003 | 8.82E-04 |
| 14 | 2.24E-03 | 8.86E-05 | 1.024 | 1.88E-03 | 1.05E-04 | 1.025 | 1.85E-03 | 1.01E-04 | 1.030 | 1.15E-03 | 0.000 | 0.000 | 1.01E-03 | 0.001 | -0.006 | 7.46E-04 | 0.100 | 0.001 | 5.64E-04 |
| 15* | 2.14E-03 | 8.82E-05 | 1.036 | 1.85E-03 | 1.04E-04 | 1.029 | 1.95E-03 | 9.86E-05 | 1.027 | 1.01E-03 | 0.000 | 0.000 | 1.06E-03 | 0.005 | -0.012 | 8.08E-04 | 0.084 | 0.002 | 8.08E-04 |
| 16 | 2.35E-03 | 8.55E-05 | 1.020 | 1.89E-03 | 1.00E-04 | 1.030 | 1.96E-03 | 9.39E-05 | 1.035 | 1.14E-03 | 0.000 | 0.001 | 1.14E-03 | 0.006 | -0.008 | 8.24E-04 | 0.106 | 0.001 | 8.24E-04 |

| **Table S2.** Parameter estimates for 16 independent MiXeR runs for the "ring" simulated scenario.  The first column show the index of the optimization run (1-16), with the run having the smallest deviation from the median overlap pattern (as described in the methods section) marked with asterisk. Other columns show univariate polygenicities ($\pi_{1}^{u},\pi_{2}^{u},\pi_{3}^{u}$), discoverabilities ($\sigma_{1},\sigma_{2},\sigma_{3}$) and residual variances ($\sigma_{01},\sigma_{02},\sigma_{03}$) for Trait 1, Trait 2 and Trait 3 respectively; pairwise (bivariate) genetic overlaps ($\pi_{12}^{b},\pi_{13}^{b},\pi_{23}^{b}$), correlations of effect sizes within each of the three pairwise overlaps ($\rho_{12},\rho_{13},\rho_{23}$) and correlations between residuals ($\rho_{012},\rho_{013},\rho_{023}$) for Trait 1 and Trait 2, Trait 1 and Trait 3 and Trait 2 and Trait 3 pairs respectively; the genetic overlap between all three phenotypes ($\pi_{123}$). | | | | | | | | | | | | | | | | | | | |
| --- | --- | --- | --- | --- | --- | --- | --- | --- | --- | --- | --- | --- | --- | --- | --- | --- | --- | --- | --- |
| Index | $\pi_{1}^{u}$ | $\sigma_{2}$ | $\sigma_{01}$ | $\pi_{2}^{u}$ | $\sigma_{2}$ | $\sigma_{02}$ | $\pi_{3}^{u}$ | $\sigma_{3}$ | $\sigma_{03}$ | $\pi_{12}^{b}$ | $\rho_{12}$ | $\rho_{012}$ | $\pi_{13}^{b}$ | $\rho_{13}$ | $\rho_{013}$ | $\pi_{23}^{b}$ | $\rho_{23}$ | $\rho_{023}$ | $\pi_{123}$ |
| 1 | 2.25E-03 | 8.67E-05 | 1.026 | 2.02E-03 | 9.59E-05 | 1.023 | 1.92E-03 | 9.66E-05 | 1.033 | 1.25E-03 | 0.000 | 0.000 | 9.00E-04 | 0.002 | -0.005 | 7.97E-04 | -0.001 | 0.009 | 2.57E-05 |
| 2 | 2.47E-03 | 8.17E-05 | 1.017 | 2.01E-03 | 9.97E-05 | 1.016 | 2.03E-03 | 9.53E-05 | 1.025 | 1.23E-03 | -0.001 | 0.005 | 1.03E-03 | 0.000 | 0.000 | 1.16E-03 | 0.000 | 0.000 | 3.74E-04 |
| 3 | 2.36E-03 | 8.46E-05 | 1.020 | 1.78E-03 | 1.12E-04 | 1.018 | 1.94E-03 | 9.96E-05 | 1.027 | 1.13E-03 | -0.005 | 0.006 | 1.01E-03 | 0.001 | -0.004 | 1.05E-03 | -0.003 | 0.004 | 3.93E-04 |
| 4 | 2.26E-03 | 8.64E-05 | 1.024 | 1.94E-03 | 1.04E-04 | 1.015 | 2.00E-03 | 9.60E-05 | 1.026 | 1.21E-03 | 0.000 | 0.000 | 1.00E-03 | 0.001 | -0.002 | 1.04E-03 | -0.004 | 0.006 | 3.06E-04 |
| 5* | 2.22E-03 | 8.57E-05 | 1.029 | 1.88E-03 | 1.09E-04 | 1.012 | 1.92E-03 | 9.95E-05 | 1.027 | 1.10E-03 | -0.001 | 0.007 | 9.59E-04 | 0.000 | 0.000 | 1.08E-03 | 0.000 | 0.005 | 3.00E-04 |
| 6 | 2.32E-03 | 8.55E-05 | 1.023 | 1.88E-03 | 1.04E-04 | 1.024 | 1.90E-03 | 9.80E-05 | 1.034 | 1.23E-03 | -0.004 | 0.005 | 1.04E-03 | 0.001 | -0.005 | 1.14E-03 | -0.002 | 0.006 | 4.89E-04 |
| 7 | 2.20E-03 | 8.61E-05 | 1.032 | 1.95E-03 | 1.05E-04 | 1.012 | 2.07E-03 | 9.40E-05 | 1.021 | 1.26E-03 | -0.004 | 0.006 | 9.28E-04 | 0.003 | -0.004 | 1.11E-03 | -0.004 | 0.005 | 4.21E-04 |
| 8 | 2.33E-03 | 8.28E-05 | 1.026 | 1.94E-03 | 1.04E-04 | 1.018 | 2.05E-03 | 9.23E-05 | 1.031 | 1.12E-03 | 0.000 | 0.000 | 1.08E-03 | 0.000 | 0.000 | 1.07E-03 | -0.008 | 0.008 | 2.52E-04 |
| 9 | 2.24E-03 | 8.76E-05 | 1.024 | 1.85E-03 | 1.06E-04 | 1.021 | 1.97E-03 | 9.71E-05 | 1.027 | 1.12E-03 | -0.002 | 0.003 | 8.10E-04 | 0.001 | -0.005 | 1.01E-03 | -0.002 | 0.004 | 2.78E-04 |
| 10 | 2.23E-03 | 8.70E-05 | 1.027 | 1.84E-03 | 1.10E-04 | 1.016 | 2.11E-03 | 9.38E-05 | 1.021 | 1.12E-03 | -0.003 | 0.006 | 9.72E-04 | 0.002 | -0.004 | 1.09E-03 | -0.001 | 0.003 | 3.62E-04 |
| 11 | 2.27E-03 | 8.57E-05 | 1.026 | 1.87E-03 | 1.07E-04 | 1.019 | 1.89E-03 | 1.01E-04 | 1.028 | 1.17E-03 | -0.004 | 0.007 | 8.28E-04 | 0.002 | -0.005 | 9.69E-04 | 0.000 | 0.001 | 2.74E-04 |
| 12 | 2.15E-03 | 8.71E-05 | 1.034 | 1.93E-03 | 1.02E-04 | 1.019 | 2.05E-03 | 9.41E-05 | 1.025 | 1.24E-03 | -0.001 | 0.005 | 9.36E-04 | 0.000 | 0.000 | 1.11E-03 | -0.004 | 0.006 | 4.25E-04 |
| 13 | 2.26E-03 | 8.65E-05 | 1.023 | 1.82E-03 | 1.07E-04 | 1.024 | 1.93E-03 | 9.78E-05 | 1.029 | 1.16E-03 | -0.005 | 0.008 | 9.08E-04 | 0.002 | -0.005 | 9.69E-04 | -0.002 | 0.004 | 3.09E-04 |
| 14 | 2.28E-03 | 8.92E-05 | 1.021 | 1.99E-03 | 1.02E-04 | 1.013 | 2.00E-03 | 9.81E-05 | 1.024 | 1.11E-03 | -0.003 | 0.003 | 9.36E-04 | 0.002 | -0.005 | 1.05E-03 | 0.000 | 0.000 | 1.63E-04 |
| 15 | 2.15E-03 | 8.96E-05 | 1.028 | 1.99E-03 | 9.90E-05 | 1.016 | 1.92E-03 | 1.01E-04 | 1.026 | 1.16E-03 | -0.006 | 0.007 | 9.21E-04 | 0.000 | 0.000 | 1.05E-03 | -0.003 | 0.006 | 2.22E-04 |
| 16 | 2.19E-03 | 8.64E-05 | 1.032 | 1.88E-03 | 1.06E-04 | 1.017 | 1.96E-03 | 9.64E-05 | 1.031 | 1.14E-03 | -0.002 | 0.006 | 9.85E-04 | 0.002 | -0.005 | 1.09E-03 | -0.002 | 0.008 | 3.49E-04 |

| **Table S3.** Parameter estimates for 16 independent MiXeR runs for the "equilibrium" simulated scenario.  The first column show the index of the optimization run (1-16), with the run having the smallest deviation from the median overlap pattern (as described in the methods section) marked with asterisk. Other columns show univariate polygenicities ($\pi_{1}^{u},\pi_{2}^{u},\pi_{3}^{u}$), discoverabilities ($\sigma_{1},\sigma_{2},\sigma_{3}$) and residual variances ($\sigma_{01},\sigma_{02},\sigma_{03}$) for Trait 1, Trait 2 and Trait 3 respectively; pairwise (bivariate) genetic overlaps ($\pi_{12}^{b},\pi_{13}^{b},\pi_{23}^{b}$), correlations of effect sizes within each of the three pairwise overlaps ($\rho_{12},\rho_{13},\rho_{23}$) and correlations between residuals ($\rho_{012},\rho_{013},\rho_{023}$) for Trait 1 and Trait 2, Trait 1 and Trait 3 and Trait 2 and Trait 3 pairs respectively; the genetic overlap between all three phenotypes ($\pi_{123}$). | | | | | | | | | | | | | | | | | | | |
| --- | --- | --- | --- | --- | --- | --- | --- | --- | --- | --- | --- | --- | --- | --- | --- | --- | --- | --- | --- |
| Index | $\pi_{1}^{u}$ | $\sigma_{2}$ | $\sigma_{01}$ | $\pi_{2}^{u}$ | $\sigma_{2}$ | $\sigma_{02}$ | $\pi_{3}^{u}$ | $\sigma_{3}$ | $\sigma_{03}$ | $\pi_{12}^{b}$ | $\rho_{12}$ | $\rho_{012}$ | $\pi_{13}^{b}$ | $\rho_{13}$ | $\rho_{013}$ | $\pi_{23}^{b}$ | $\rho_{23}$ | $\rho_{023}$ | $\pi_{123}$ |
| 1 | 1.99E-03 | 9.14E-05 | 1.035 | 1.95E-03 | 9.73E-05 | 1.027 | 2.02E-03 | 9.22E-05 | 1.030 | 1.01E-03 | 0.002 | -0.003 | 1.26E-03 | 0.002 | -0.003 | 9.45E-04 | -0.010 | 0.009 | 5.29E-04 |
| 2 | 2.11E-03 | 8.61E-05 | 1.032 | 2.02E-03 | 9.24E-05 | 1.030 | 1.97E-03 | 9.66E-05 | 1.026 | 1.04E-03 | 0.002 | -0.006 | 1.19E-03 | 0.002 | -0.006 | 8.77E-04 | -0.002 | 0.005 | 5.22E-04 |
| 3 | 2.04E-03 | 8.97E-05 | 1.034 | 2.12E-03 | 8.84E-05 | 1.027 | 2.07E-03 | 9.29E-05 | 1.026 | 1.15E-03 | 0.002 | -0.007 | 1.17E-03 | 0.000 | 0.000 | 1.06E-03 | -0.005 | 0.005 | 6.30E-04 |
| 4 | 2.10E-03 | 8.69E-05 | 1.035 | 2.06E-03 | 9.29E-05 | 1.027 | 1.97E-03 | 9.26E-05 | 1.034 | 1.01E-03 | 0.002 | -0.007 | 1.30E-03 | 0.002 | -0.004 | 9.15E-04 | -0.003 | 0.004 | 8.37E-04 |
| 5 | 2.13E-03 | 8.52E-05 | 1.032 | 2.03E-03 | 8.94E-05 | 1.036 | 1.88E-03 | 9.98E-05 | 1.029 | 1.24E-03 | 0.002 | -0.007 | 1.32E-03 | 0.001 | -0.001 | 7.70E-04 | -0.004 | 0.006 | 5.44E-04 |
| 6 | 2.09E-03 | 8.89E-05 | 1.030 | 1.98E-03 | 8.99E-05 | 1.038 | 2.12E-03 | 9.16E-05 | 1.022 | 1.05E-03 | 0.002 | -0.004 | 1.31E-03 | 0.000 | 0.000 | 8.53E-04 | 0.000 | 0.000 | 3.72E-04 |
| 7 | 2.16E-03 | 8.35E-05 | 1.034 | 1.96E-03 | 9.13E-05 | 1.038 | 2.17E-03 | 8.65E-05 | 1.031 | 1.09E-03 | 0.001 | -0.001 | 1.40E-03 | 0.001 | -0.003 | 9.22E-04 | 0.000 | 0.000 | 8.60E-04 |
| 8 | 2.06E-03 | 8.57E-05 | 1.039 | 2.00E-03 | 8.72E-05 | 1.042 | 1.92E-03 | 9.77E-05 | 1.033 | 1.18E-03 | 0.001 | -0.007 | 1.18E-03 | 0.001 | -0.001 | 9.62E-04 | -0.005 | 0.005 | 7.60E-04 |
| 9 | 1.98E-03 | 9.36E-05 | 1.030 | 2.12E-03 | 8.99E-05 | 1.025 | 2.11E-03 | 9.21E-05 | 1.024 | 1.18E-03 | 0.003 | -0.008 | 1.37E-03 | 0.001 | -0.002 | 1.03E-03 | -0.005 | 0.005 | 7.87E-04 |
| 10 | 1.87E-03 | 9.55E-05 | 1.040 | 2.02E-03 | 9.03E-05 | 1.035 | 2.06E-03 | 9.15E-05 | 1.031 | 9.49E-04 | 0.002 | -0.007 | 1.15E-03 | 0.000 | -0.001 | 1.02E-03 | -0.010 | 0.010 | 4.94E-04 |
| 11* | 2.00E-03 | 8.86E-05 | 1.039 | 2.05E-03 | 9.12E-05 | 1.026 | 1.99E-03 | 9.46E-05 | 1.028 | 1.14E-03 | 0.002 | -0.005 | 1.23E-03 | 0.000 | 0.000 | 8.84E-04 | -0.003 | 0.006 | 5.56E-04 |
| 12 | 2.10E-03 | 8.70E-05 | 1.034 | 2.07E-03 | 9.00E-05 | 1.027 | 2.03E-03 | 9.31E-05 | 1.030 | 1.03E-03 | 0.003 | -0.008 | 1.30E-03 | 0.005 | -0.010 | 8.93E-04 | -0.003 | 0.006 | 5.21E-04 |
| 13 | 2.03E-03 | 8.82E-05 | 1.037 | 2.07E-03 | 9.01E-05 | 1.030 | 2.07E-03 | 9.15E-05 | 1.031 | 1.14E-03 | 0.001 | -0.006 | 1.37E-03 | 0.002 | -0.006 | 9.13E-04 | -0.002 | 0.004 | 7.33E-04 |
| 14 | 2.06E-03 | 8.56E-05 | 1.041 | 2.01E-03 | 9.00E-05 | 1.034 | 2.15E-03 | 9.10E-05 | 1.023 | 1.12E-03 | 0.006 | -0.008 | 1.31E-03 | 0.002 | -0.008 | 9.41E-04 | -0.003 | 0.003 | 5.74E-04 |
| 15 | 2.04E-03 | 8.67E-05 | 1.038 | 2.05E-03 | 8.85E-05 | 1.033 | 2.12E-03 | 9.33E-05 | 1.020 | 1.16E-03 | 0.002 | -0.007 | 1.24E-03 | 0.000 | -0.004 | 9.75E-04 | -0.010 | 0.010 | 7.01E-04 |
| 16 | 2.13E-03 | 8.48E-05 | 1.034 | 2.23E-03 | 8.62E-05 | 1.023 | 1.92E-03 | 9.88E-05 | 1.028 | 1.13E-03 | 0.005 | -0.013 | 1.24E-03 | 0.000 | 0.000 | 9.26E-04 | -0.004 | 0.003 | 5.97E-04 |

| **Table S4.** Parameter estimates for 16 independent MiXeR runs for genetic generalized epilepsy (GGE), irritable bowel syndrome (IBS) and attention deficit hyperactivity disorder (ADHD).  The first column show the index of the optimization run (1-16), with the run having the smallest deviation from the median overlap pattern (as described in the methods section) marked with asterisk. Other columns show univariate polygenicities ($\pi_{1}^{u},\pi_{2}^{u},\pi_{3}^{u}$), discoverabilities ($\sigma_{1},\sigma_{2},\sigma_{3}$) and residual variances ($\sigma_{01},\sigma_{02},\sigma_{03}$) for GGE, IBS and ADHD respectively; pairwise (bivariate) genetic overlaps ($\pi_{12}^{b},\pi_{13}^{b},\pi_{23}^{b}$), correlations of effect sizes within each of the three pairwise overlaps ($\rho_{12},\rho_{13},\rho_{23}$) and correlations between residuals ($\rho_{012},\rho_{013},\rho_{023}$) for GGE and IBS, GGE and ADHD and IBS and ADHD pairs respectively; the genetic overlap between all three phenotypes ($\pi_{123}$). | | | | | | | | | | | | | | | | | | | |
| --- | --- | --- | --- | --- | --- | --- | --- | --- | --- | --- | --- | --- | --- | --- | --- | --- | --- | --- | --- |
| Index | $\pi_{1}^{u}$ | $\sigma_{2}$ | $\sigma_{01}$ | $\pi_{2}^{u}$ | $\sigma_{2}$ | $\sigma_{02}$ | $\pi_{3}^{u}$ | $\sigma_{3}$ | $\sigma_{03}$ | $\pi_{12}^{b}$ | $\rho_{12}$ | $\rho_{012}$ | $\pi_{13}^{b}$ | $\rho_{13}$ | $\rho_{013}$ | $\pi_{23}^{b}$ | $\rho_{23}$ | $\rho_{023}$ | $\pi_{123}$ |
| 1 | 1.55E-03 | 1.90E-04 | 1.083 | 2.01E-03 | 2.09E-05 | 1.017 | 2.96E-03 | 2.83E-05 | 1.121 | 1.55E-03 | 0.000 | 0.000 | 1.36E-03 | 0.096 | 0.013 | 1.67E-03 | 0.182 | 0.022 | 1.36E-03 |
| 2 | 1.31E-03 | 2.08E-04 | 1.095 | 2.40E-03 | 1.85E-05 | 1.015 | 2.91E-03 | 2.85E-05 | 1.124 | 1.21E-03 | 0.000 | 0.000 | 2.75E-04 | 0.614 | 0.012 | 1.42E-03 | 0.442 | 0.014 | 2.37E-04 |
| 3 | 1.57E-03 | 1.85E-04 | 1.084 | 2.96E-03 | 1.46E-05 | 1.017 | 2.92E-03 | 2.81E-05 | 1.123 | 1.57E-03 | 0.000 | 0.000 | 6.68E-04 | 0.320 | 0.011 | 1.60E-03 | 0.410 | 0.019 | 6.68E-04 |
| 4 | 1.43E-03 | 2.03E-04 | 1.089 | 2.44E-03 | 1.67E-05 | 1.023 | 2.96E-03 | 2.80E-05 | 1.121 | 1.43E-03 | 0.000 | 0.000 | 1.12E-03 | 0.176 | 0.010 | 1.52E-03 | 0.410 | 0.019 | 1.12E-03 |
| 5 | 1.43E-03 | 1.92E-04 | 1.093 | 1.91E-03 | 2.13E-05 | 1.023 | 3.15E-03 | 2.60E-05 | 1.123 | 1.22E-03 | 0.000 | 0.000 | 1.10E-03 | 0.140 | 0.014 | 9.61E-04 | 0.629 | 0.017 | 8.85E-04 |
| 6 | 1.41E-03 | 2.02E-04 | 1.091 | 2.36E-03 | 1.80E-05 | 1.020 | 3.13E-03 | 2.67E-05 | 1.119 | 1.41E-03 | -0.104 | 0.001 | 1.33E-03 | 0.077 | 0.014 | 1.44E-03 | 0.476 | 0.014 | 1.33E-03 |
| 7* | 1.44E-03 | 1.91E-04 | 1.095 | 2.27E-03 | 1.88E-05 | 1.017 | 3.05E-03 | 2.68E-05 | 1.122 | 1.30E-03 | 0.000 | 0.000 | 1.22E-03 | 0.143 | 0.011 | 1.12E-03 | 0.521 | 0.016 | 1.08E-03 |
| 8 | 1.31E-03 | 2.10E-04 | 1.096 | 1.98E-03 | 2.13E-05 | 1.016 | 3.22E-03 | 2.70E-05 | 1.110 | 1.04E-03 | 0.000 | 0.000 | 9.99E-04 | 0.150 | 0.012 | 9.12E-04 | 0.607 | 0.015 | 7.37E-04 |
| 9 | 1.58E-03 | 1.87E-04 | 1.084 | 2.17E-03 | 1.90E-05 | 1.023 | 3.02E-03 | 2.76E-05 | 1.120 | 1.58E-03 | 0.000 | 0.001 | 1.07E-03 | 0.197 | 0.011 | 1.09E-03 | 0.514 | 0.019 | 1.07E-03 |
| 10 | 1.42E-03 | 2.03E-04 | 1.089 | 2.69E-03 | 1.61E-05 | 1.014 | 2.77E-03 | 2.91E-05 | 1.123 | 1.34E-03 | 0.000 | 0.000 | 1.18E-03 | 0.163 | 0.007 | 2.48E-03 | 0.161 | 0.020 | 1.16E-03 |
| 11 | 1.55E-03 | 1.82E-04 | 1.093 | 2.47E-03 | 1.81E-05 | 1.017 | 2.89E-03 | 2.87E-05 | 1.121 | 1.23E-03 | 0.000 | 0.000 | 1.30E-03 | 0.100 | 0.014 | 2.26E-03 | 0.181 | 0.023 | 1.02E-03 |
| 12 | 1.45E-03 | 1.96E-04 | 1.090 | 3.29E-03 | 1.31E-05 | 1.018 | 2.82E-03 | 2.83E-05 | 1.125 | 1.45E-03 | 0.000 | 0.000 | 1.29E-03 | 0.124 | 0.015 | 1.37E-03 | 0.493 | 0.021 | 1.29E-03 |
| 13 | 1.25E-03 | 2.16E-04 | 1.099 | 3.42E-03 | 1.26E-05 | 1.016 | 2.90E-03 | 2.88E-05 | 1.121 | 1.25E-03 | 0.000 | 0.000 | 1.04E-03 | 0.151 | 0.010 | 1.50E-03 | 0.579 | 0.012 | 1.04E-03 |
| 14 | 1.27E-03 | 2.11E-04 | 1.098 | 1.59E-03 | 2.41E-05 | 1.025 | 3.16E-03 | 2.61E-05 | 1.125 | 1.08E-03 | 0.000 | 0.000 | 1.05E-03 | 0.121 | 0.015 | 9.70E-04 | 0.583 | 0.017 | 8.59E-04 |
| 15 | 1.20E-03 | 2.24E-04 | 1.099 | 2.88E-03 | 1.41E-05 | 1.020 | 3.13E-03 | 2.69E-05 | 1.116 | 1.17E-03 | -0.105 | 0.003 | 9.72E-04 | 0.149 | 0.012 | 1.00E-03 | 0.735 | 0.017 | 9.41E-04 |
| 16 | 1.62E-03 | 1.78E-04 | 1.088 | 2.52E-03 | 1.71E-05 | 1.015 | 3.01E-03 | 2.76E-05 | 1.122 | 9.04E-04 | 0.000 | 0.000 | 2.86E-04 | 0.883 | 0.010 | 1.86E-03 | 0.345 | 0.007 | 2.47E-04 |

| **Table S5.** Parameter estimates for 16 independent MiXeR runs for type 2 diabetes (T2D), estimated glomerular filtration rate (eGFR) and high-density lipoprotein (HDL).  The first column show the index of the optimization run (1-16), with the run having the smallest deviation from the median overlap pattern (as described in the methods section) marked with asterisk. Other columns show univariate polygenicities ($\pi_{1}^{u},\pi_{2}^{u},\pi_{3}^{u}$), discoverabilities ($\sigma_{1},\sigma_{2},\sigma_{3}$) and residual variances ($\sigma_{01},\sigma_{02},\sigma_{03}$) for T2D, eGFR and HDL respectively; pairwise (bivariate) genetic overlaps ($\pi_{12}^{b},\pi_{13}^{b},\pi_{23}^{b}$), correlations of effect sizes within each of the three pairwise overlaps ($\rho_{12},\rho_{13},\rho_{23}$) and correlations between residuals ($\rho_{012},\rho_{013},\rho_{023}$) for T2D and eGFR, T2D and HDL and eGFR and HDL pairs respectively; the genetic overlap between all three phenotypes ($\pi_{123}$). | | | | | | | | | | | | | | | | | | | |
| --- | --- | --- | --- | --- | --- | --- | --- | --- | --- | --- | --- | --- | --- | --- | --- | --- | --- | --- | --- |
| Index | $\pi_{1}^{u}$ | $\sigma_{2}$ | $\sigma_{01}$ | $\pi_{2}^{u}$ | $\sigma_{2}$ | $\sigma_{02}$ | $\pi_{3}^{u}$ | $\sigma_{3}$ | $\sigma_{03}$ | $\pi_{12}^{b}$ | $\rho_{12}$ | $\rho_{012}$ | $\pi_{13}^{b}$ | $\rho_{13}$ | $\rho_{013}$ | $\pi_{23}^{b}$ | $\rho_{23}$ | $\rho_{023}$ | $\pi_{123}$ |
| 1 | 9.12E-04 | 1.08E-04 | 1.061 | 6.02E-04 | 7.95E-05 | 1.077 | 6.30E-04 | 1.51E-04 | 1.358 | 2.68E-04 | 0.116 | 0.006 | 4.61E-04 | -0.526 | -0.187 | 2.18E-04 | 0.000 | 0.001 | 6.08E-05 |
| 2 | 7.97E-04 | 1.25E-04 | 1.070 | 6.56E-04 | 7.49E-05 | 1.068 | 7.76E-04 | 1.24E-04 | 1.292 | 2.39E-04 | 0.104 | 0.002 | 4.17E-04 | -0.700 | -0.173 | 2.87E-04 | 0.145 | 0.000 | 2.39E-04 |
| 3 | 8.41E-04 | 1.08E-04 | 1.079 | 6.09E-04 | 7.87E-05 | 1.080 | 7.40E-04 | 1.32E-04 | 1.307 | 2.71E-04 | 0.133 | 0.000 | 4.18E-04 | -0.386 | -0.214 | 1.06E-04 | 0.000 | 0.000 | 5.43E-05 |
| 4 | 1.10E-03 | 9.48E-05 | 1.042 | 6.35E-04 | 7.50E-05 | 1.075 | 7.68E-04 | 1.29E-04 | 1.298 | 3.38E-04 | 0.215 | 0.000 | 7.01E-04 | -0.311 | -0.219 | 3.52E-04 | 0.152 | 0.000 | 3.38E-04 |
| 5* | 8.50E-04 | 1.12E-04 | 1.069 | 5.85E-04 | 8.30E-05 | 1.078 | 7.20E-04 | 1.33E-04 | 1.319 | 2.99E-04 | 0.152 | 0.000 | 3.92E-04 | -0.741 | -0.149 | 2.28E-04 | 0.153 | 0.000 | 2.28E-04 |
| 6 | 8.29E-04 | 1.18E-04 | 1.068 | 5.71E-04 | 8.69E-05 | 1.079 | 7.24E-04 | 1.31E-04 | 1.335 | 2.35E-04 | 0.133 | 0.002 | 3.65E-04 | -0.688 | -0.219 | 1.98E-04 | -0.074 | 0.078 | 1.98E-04 |
| 7 | 7.93E-04 | 1.15E-04 | 1.080 | 7.08E-04 | 7.01E-05 | 1.064 | 5.48E-04 | 1.79E-04 | 1.382 | 2.82E-04 | 0.104 | 0.001 | 3.80E-04 | -0.537 | -0.212 | 2.46E-04 | 0.075 | 0.009 | 9.20E-05 |
| 8 | 7.77E-04 | 1.26E-04 | 1.067 | 5.94E-04 | 8.52E-05 | 1.072 | 5.81E-04 | 1.84E-04 | 1.337 | 2.56E-04 | 0.001 | -0.002 | 3.28E-04 | -0.675 | -0.229 | 1.04E-04 | 0.224 | 0.000 | 5.34E-05 |
| 9 | 8.73E-04 | 1.15E-04 | 1.057 | 6.27E-04 | 7.70E-05 | 1.078 | 7.56E-04 | 1.32E-04 | 1.306 | 3.10E-04 | 0.103 | 0.003 | 4.34E-04 | -0.639 | -0.191 | 2.60E-04 | 0.141 | 0.008 | 1.18E-04 |
| 10 | 7.95E-04 | 1.22E-04 | 1.066 | 6.32E-04 | 7.70E-05 | 1.071 | 7.65E-04 | 1.27E-04 | 1.298 | 3.24E-04 | 0.162 | 0.000 | 4.58E-04 | -0.305 | -0.229 | 2.41E-04 | 0.151 | 0.000 | 2.41E-04 |
| 11 | 8.88E-04 | 1.08E-04 | 1.067 | 6.64E-04 | 7.61E-05 | 1.061 | 8.04E-04 | 1.21E-04 | 1.292 | 2.38E-04 | 0.224 | 0.000 | 6.17E-04 | -0.304 | -0.221 | 2.79E-04 | 0.145 | 0.000 | 2.38E-04 |
| 12 | 9.58E-04 | 1.04E-04 | 1.049 | 6.75E-04 | 7.22E-05 | 1.079 | 6.95E-04 | 1.40E-04 | 1.325 | 1.14E-04 | 0.191 | 0.000 | 3.68E-04 | -0.624 | -0.228 | 2.53E-04 | 0.136 | 0.000 | 5.79E-05 |
| 13 | 7.78E-04 | 1.25E-04 | 1.068 | 5.75E-04 | 8.73E-05 | 1.076 | 6.08E-04 | 1.48E-04 | 1.386 | 2.33E-04 | 0.144 | 0.000 | 2.32E-04 | -0.754 | -0.212 | 1.19E-04 | 0.217 | 0.001 | 3.04E-05 |
| 14 | 1.04E-03 | 9.62E-05 | 1.051 | 6.19E-04 | 7.95E-05 | 1.076 | 6.05E-04 | 1.71E-04 | 1.348 | 2.72E-04 | 0.073 | 0.002 | 5.14E-04 | -0.519 | -0.179 | 1.84E-04 | 0.225 | 0.000 | 1.84E-04 |
| 15 | 8.44E-04 | 1.08E-04 | 1.079 | 6.19E-04 | 8.11E-05 | 1.069 | 5.96E-04 | 1.60E-04 | 1.374 | 2.95E-04 | 0.124 | 0.002 | 2.08E-04 | -0.894 | -0.222 | 2.20E-04 | 0.193 | 0.001 | 2.08E-04 |
| 16 | 7.26E-04 | 1.28E-04 | 1.077 | 5.60E-04 | 8.57E-05 | 1.083 | 7.67E-04 | 1.25E-04 | 1.305 | 2.43E-04 | 0.114 | 0.000 | 2.19E-04 | -0.955 | -0.216 | 2.37E-05 | 0.442 | 0.000 | 1.00E-06 |

| **Table S6.** Parameter estimates for 16 independent MiXeR runs for ulcerative colitis (UC), multiple sclerosis (MS) and psoriasis (PS).  The first column show the index of the optimization run (1-16), with the run having the smallest deviation from the median overlap pattern (as described in the methods section) marked with asterisk. Other columns show univariate polygenicities ($\pi_{1}^{u},\pi_{2}^{u},\pi_{3}^{u}$), discoverabilities ($\sigma_{1},\sigma_{2},\sigma_{3}$) and residual variances ($\sigma_{01},\sigma_{02},\sigma_{03}$) for UC, MS and PS respectively; pairwise (bivariate) genetic overlaps ($\pi_{12}^{b},\pi_{13}^{b},\pi_{23}^{b}$), correlations of effect sizes within each of the three pairwise overlaps ($\rho_{12},\rho_{13},\rho_{23}$) and correlations between residuals ($\rho_{012},\rho_{013},\rho_{023}$) for UC and MS, UC and PS and MS and PS pairs respectively; the genetic overlap between all three phenotypes ($\pi_{123}$). | | | | | | | | | | | | | | | | | | | |
| --- | --- | --- | --- | --- | --- | --- | --- | --- | --- | --- | --- | --- | --- | --- | --- | --- | --- | --- | --- |
| Index | $\pi_{1}^{u}$ | $\sigma_{2}$ | $\sigma_{01}$ | $\pi_{2}^{u}$ | $\sigma_{2}$ | $\sigma_{02}$ | $\pi_{3}^{u}$ | $\sigma_{3}$ | $\sigma_{03}$ | $\pi_{12}^{b}$ | $\rho_{12}$ | $\rho_{012}$ | $\pi_{13}^{b}$ | $\rho_{13}$ | $\rho_{013}$ | $\pi_{23}^{b}$ | $\rho_{23}$ | $\rho_{023}$ | $\pi_{123}$ |
| 1 | 1.69E-04 | 1.10E-03 | 1.126 | 3.60E-04 | 6.46E-04 | 1.040 | 1.44E-04 | 7.68E-04 | 1.101 | 1.37E-04 | 0.000 | 0.000 | 8.44E-05 | 0.000 | 0.000 | 1.27E-04 | 0.127 | -0.001 | 8.44E-05 |
| 2 | 1.89E-04 | 1.04E-03 | 1.123 | 2.57E-04 | 8.80E-04 | 1.055 | 1.94E-04 | 6.65E-04 | 1.081 | 1.49E-04 | 0.000 | 0.001 | 1.12E-04 | 0.292 | 0.001 | 1.20E-04 | 0.105 | 0.002 | 1.05E-04 |
| 3 | 1.94E-04 | 1.10E-03 | 1.117 | 2.39E-04 | 8.88E-04 | 1.059 | 1.34E-04 | 8.00E-04 | 1.105 | 1.09E-04 | 0.000 | 0.000 | 9.41E-05 | 0.000 | 0.000 | 9.22E-05 | 0.000 | 0.000 | 8.49E-05 |
| 4 | 1.98E-04 | 9.90E-04 | 1.123 | 3.35E-04 | 6.94E-04 | 1.046 | 1.26E-04 | 9.20E-04 | 1.097 | 1.50E-04 | -0.001 | 0.004 | 9.66E-05 | 0.000 | 0.000 | 1.17E-04 | 0.217 | 0.000 | 9.66E-05 |
| 5* | 1.71E-04 | 1.11E-03 | 1.123 | 2.72E-04 | 8.22E-04 | 1.058 | 1.29E-04 | 8.28E-04 | 1.100 | 1.23E-04 | 0.000 | 0.000 | 8.56E-05 | 0.000 | 0.000 | 1.02E-04 | 0.105 | 0.005 | 8.12E-05 |
| 6 | 1.78E-04 | 1.11E-03 | 1.127 | 2.70E-04 | 8.24E-04 | 1.053 | 9.77E-05 | 1.04E-03 | 1.110 | 1.27E-04 | 0.000 | 0.000 | 6.64E-05 | 0.002 | -0.004 | 8.65E-05 | 0.103 | -0.003 | 6.64E-05 |
| 7 | 1.84E-04 | 1.02E-03 | 1.126 | 2.83E-04 | 8.59E-04 | 1.051 | 7.22E-05 | 1.29E-03 | 1.117 | 1.29E-04 | 0.001 | -0.003 | 5.10E-05 | 0.000 | 0.000 | 6.71E-05 | 0.139 | -0.002 | 5.10E-05 |
| 8 | 1.75E-04 | 1.09E-03 | 1.129 | 2.88E-04 | 8.40E-04 | 1.049 | 1.95E-04 | 6.12E-04 | 1.092 | 1.26E-04 | 0.107 | 0.001 | 1.04E-04 | 0.103 | 0.001 | 1.41E-04 | 0.118 | 0.003 | 1.04E-04 |
| 9 | 2.55E-04 | 8.64E-04 | 1.104 | 3.18E-04 | 7.38E-04 | 1.047 | 6.71E-05 | 1.65E-03 | 1.107 | 1.70E-04 | 0.000 | 0.000 | 6.71E-05 | 0.000 | 0.000 | 6.49E-05 | 0.114 | 0.006 | 6.49E-05 |
| 10 | 2.03E-04 | 9.67E-04 | 1.122 | 2.91E-04 | 7.82E-04 | 1.048 | 1.11E-04 | 1.03E-03 | 1.101 | 1.60E-04 | 0.000 | 0.000 | 8.74E-05 | 0.003 | -0.006 | 9.27E-05 | 0.156 | 0.001 | 8.74E-05 |
| 11 | 1.91E-04 | 1.06E-03 | 1.122 | 2.51E-04 | 9.60E-04 | 1.051 | 2.08E-04 | 5.73E-04 | 1.085 | 1.29E-04 | 0.000 | 0.000 | 1.35E-04 | 0.035 | 0.001 | 1.31E-04 | 0.000 | 0.000 | 1.10E-04 |
| 12 | 1.60E-04 | 1.22E-03 | 1.131 | 2.69E-04 | 8.85E-04 | 1.047 | 7.15E-05 | 1.40E-03 | 1.112 | 1.13E-04 | 0.000 | 0.004 | 6.15E-05 | 0.000 | 0.000 | 6.98E-05 | 0.192 | 0.002 | 6.15E-05 |
| 13 | 1.69E-04 | 1.15E-03 | 1.119 | 2.42E-04 | 8.87E-04 | 1.061 | 1.33E-04 | 8.33E-04 | 1.095 | 1.08E-04 | -0.002 | 0.003 | 6.99E-05 | 0.153 | 0.001 | 8.35E-05 | 0.205 | 0.000 | 5.87E-05 |
| 14 | 2.17E-04 | 9.06E-04 | 1.123 | 3.46E-04 | 6.89E-04 | 1.046 | 1.03E-04 | 1.01E-03 | 1.106 | 1.73E-04 | 0.126 | -0.003 | 7.93E-05 | 0.000 | 0.000 | 1.03E-04 | 0.063 | 0.004 | 7.93E-05 |
| 15 | 1.92E-04 | 1.00E-03 | 1.121 | 3.51E-04 | 6.44E-04 | 1.048 | 1.41E-04 | 7.71E-04 | 1.100 | 1.67E-04 | -0.007 | 0.007 | 8.82E-05 | 0.158 | -0.005 | 1.30E-04 | 0.090 | 0.003 | 8.82E-05 |
| 16 | 2.00E-04 | 1.08E-03 | 1.122 | 2.90E-04 | 8.01E-04 | 1.048 | 1.79E-04 | 6.11E-04 | 1.098 | 1.33E-04 | 0.002 | -0.005 | 1.25E-04 | 0.221 | 0.000 | 1.34E-04 | 0.168 | -0.001 | 1.17E-04 |
